# A Critical Appraisal of the Application of Frailty and Sarcopenia in the Spinal Oncology Population

**DOI:** 10.1177/21925682231207325

**Published:** 2025-01-12

**Authors:** Mark A. MacLean, Antoinette J. Charles, Miltiadis Georgiopoulos, Jackie Phinney, Raphaële Charest-Morin, Rory Goodwin, Ilya Laufer, Michael G. Fehlings, John Shin, Nicholas Dea, Laurence D. Rhines, Arjun Sahgal, Ziya Gokaslan, Byron Stephens, Alexander C. Disch, Naresh Kumar, John O’Toole, Daniel M. Sciubba, Cordula Netzer, Tony Goldschlager, Wende Gibbs, Michael H. Weber, on behalf of the AO Spine Knowledge Forum Tumor

**Affiliations:** 1 Division of Neurosurgery, Department of Surgery, 3688 Dalhousie University, Halifax, NS, Canada; 2 Spine Division, Department of Neurosurgery, 3065 Duke University, Durham, NC, USA; 3 Spine Surgery Program, Department of Surgery, 5620 McGill University, Montreal, QC, Canada; 4 W.K Kellogg Health Sciences Library, Saint John, NB, Canada; 5 Spine Surgery Institute, 8167 Vancouver General Hospital, University of British Columbia, Vancouver, BC, Canada; 6 Department of Neurosurgery, 12297 New York University Langone Health, New York, NY, USA; 7 Division of Neurosurgery and Spine Program, Department of Surgery, Toronto Western Hospital, University of Toronto, 7989 University Health Network, Toronto, ON, Canada; 8 Department of Neurosurgery, Massachusetts General Hospital, 1812 Harvard University, Boston, MA, USA; 9 Division of Surgery, Department of Neurosurgery, The University of Texas MD Anderson Cancer Centre, Houston, TX, USA; 10 71545 Sunnybrook Health Sciences Centre, Department of Radiation Oncology, University of Toronto, Toronto, ON, Canada; 11 Department of Neurosurgery, 12321 The Warren Alpert Medical School of Brown University, Providence, RI, USA; 12 Department of Orthopaedic Surgery, 12328 Vanderbilt University Medical Center, Nashville, TN, USA; 13 University Center for Orthopedics, Trauma & Plastic Surgery, 39063 University Hospital Carl Gustav Carus at the TU Dresden, Dresden, Germany; 14 Department of Orthopedic Surgery, 150744 National University Health System, Singapore; 15 Department of Neurosurgery, 2461 Rush University, Chicago, IL, USA; 16 Department of Neurosurgery, Zucker School of Medicine at Hofstra, Long Island Jewish Medical Center and North Shore University Hospital, Northwell Health, Manhasset, NY, USA; 17 Department of Spine Surgery, 30262 University Hospital of Basel, Basel, Switzerland; 18 Department of Surgery, Monash University, Melbourne, VIC, Australia; 19 Department of Neuroradiology, 115467 Barrow Neurological Institute, Phoenix, AZ, USA

**Keywords:** spine, tumor, metastatic, disease, frailty, sarcopenia

## Abstract

**Study Design:**

Systematic review and clinimetric analysis.

**Objectives:**

Frailty and sarcopenia predict worse surgical outcomes among spinal degenerative and deformity-related populations; this association is less clear in the context of spinal oncology. Here, we sought to identify frailty and sarcopenia tools applied in spinal oncology and appraise their clinimetric properties.

**Methods:**

A systematic review was conducted from January 1^st^, 2000, until June 2022. Study characteristics, frailty tools, and measures of sarcopenia were recorded. Component domains, individual items, cut-off values, and measurement techniques were collected. Clinimetric assessment was performed according to Consensus-based Standards for Health Measurement Instruments.

**Results:**

Twenty-two studies were included (42 514 patients). Seventeen studies utilized 6 frailty tools; the three most employed were the Metastatic Spine tumor Frailty Index (MSTFI), Modified Frailty Index-11 (mFI-11), and the mFI-5. Eight studies utilized measures of sarcopenia; the three most common were the L3-Total Psoas Area (TPA)/Vertebral Body Area (VBA), L3-TPA/Height^2^, and L3-Spinal Muscle Index (L3-Cross-Sectional Muscle Area/Height^2^). Frailty and sarcopenia measures lacked or had uncertain content and construct validity. Frailty measures were objective except the Johns-Hopkins Adjusted Clinical Groups. All tools were feasible except the Hospital Frailty Risk Score (HFRS). Positive predictive validity was observed for the HFRS and in select studies employing the mFI-5, MSTFI, and L3-TPA/VBA. All frailty tools had floor or ceiling effects.

**Conclusions:**

Existing tools for evaluating frailty and sarcopenia among patients undergoing surgery for spinal tumors have poor clinimetric properties. Here, we provide a pragmatic approach to utilizing existing frailty and sarcopenia tools, until more clinimetrically robust instruments are developed.

## Introduction

The NOMS (neurological, oncological, mechanical, systemic) framework was developed to provide a common language across medical and surgical disciplines and simplify the complicated decision-making process inherent to spinal oncology.^1^ Evaluation of systemic condition remains an unaddressed pillar in the framework, which vaguely advises an estimation of patients’ physical reserve and the ability to tolerate surgery. Complexity in this process largely owes to the numerous pre-operative systemic variables that influence postoperative clinical outcomes in this patient population.^2^ Given that older patients with multiple comorbidities constitute a substantial portion of patients with cancer, further research should strive to clarify the systemic component of the NOMS framework.

Frailty is an attractive surrogate for systemic condition and has been widely studied in medicine and surgery.^3,4^ Frailty may be operationalized as a phenotypic definition, or a composite index of accumulating deficits and diminished physical reserve.^4–6^ Increased frailty predicts worse clinical outcomes among patients undergoing spinal surgery for degenerative and deformity-related conditions,^3,7^ however, this association is less clear in the context of spinal oncology.^8–11^ An existing Metastatic Spinal Tumor Frailty Index was found to have poor clinimetric properties (e.g. content and predictive validity),^12^ and discrimination for clinical outcomes.^9^ Until recently, controversy existed regarding the definition and determinants of frailty among patients with spinal tumors. Consensus was attained among members of the AO Spine community, regarding a phenotypic definition of frailty and the association between 14 preoperative variables and frailty in the context of spinal metastatic disease, respectively.^13^ This represents an important first step toward the development of a clinimetrically robust tool for use in this patient population.

Sarcopenia is an alternative indicator of frailty and surrogate for systemic condition, defined as a progressive loss of skeletal muscle mass and power.^10^ Like frailty, sarcopenia has been found to predict functional decline and poor clinical outcomes in surgical populations,^14–16^ but not consistently among patients with spinal tumors.^8,17,18^ Various measurement techniques have been described, including the measurement of psoas area on axial CT imaging. Body composition analysis (BCA) expands on such traditional measurement techniques and may better capture total body sarcopenia.^19–21^ BCA may include a skeletal muscle index (SMI; paraspinal, psoas, and abdominal cross-sectional skeletal muscle quantity) and radiodensity (SMQ; skeletal muscle quality), as well as visceral and subcutaneous adiposity.^22^ Recent advancements in the utilization of artificial intelligence and automated imaging analysis across surgical disciplines may offer improved feasibility and uptake of techniques like BCA into the clinical setting; however, the association with clinical outcomes remains to be determined.^18,22,23^

Further research should clarify the role of frailty and sarcopenia as surrogates for systemic condition in the context of surgical spine oncology. Here, we sought to gain insight regarding existing knowledge gaps by reviewing existing frailty and sarcopenia tools applied in surgical spinal oncology and assessing their clinimetric properties. Our specific study objectives included: (1) to identify frailty and sarcopenia tools used in spinal oncology (primary and metastatic disease); (2) appraise the objectivity, feasibility, and clinimetric properties of these tools; and (3) to assess and summarize the application of these tools in the context of risk stratification and outcome prognostication. These findings may facilitate the development of a clinimetrically robust tool for estimating systemic condition, stratifying risk, and predicting clinical outcomes in patients undergoing surgery for spinal tumors.

## Methods

### Study Design

The AO Spine Knowledge Forum Tumor conducted a systematic review that is reported according to the 2020 Preferred Reporting Items for Systematic Reviews and Meta-Analyses statement (PRISMA checklist: Supplemental Content A).^24,25^ This study design was utilized in order to comprehensively synthesize studies applying tools toward the measurement of frailty and sarcopenia in surgical spinal oncology populations. The protocol for this study was registered with PROSPERO international prospective register for systematic reviews (registration number: CRD42022367938).^26^ This study was Institutional Review Board exempt and informed consent was not sought, given that no identifiable health information was collected and only data from published studies were incorporated.

### Search Strategy and Selection Criteria

References were identified via a comprehensive electronic search of the English literature conducted by an experienced medical librarian (JP). The search strategy was developed in Ovid MEDLINE and tested against a set of pre-identified studies before undergoing peer-review by a second medical librarian using the PRESS checklist.^27^ The search was then translated and executed across all databases used in this review (Ovid MEDLINE, Embase (Elsevier), Cochrane Central Register of Controlled Trials (Cochrane Library), and CINAHL (EBSCO). Databases were searched from January 1^st^, 2000, until June 15^th^, 2022 (date of last search). These date filters were utilized to capture emerging evidence, while accounting for evolving surgical techniques in surgical spinal oncology. This review was not funded. As such, translation services were not used and English language filters were applied when provided by the database. We searched terms relevant to surgery (neurosurgery, orthopedic surgery, operative, perioperative), spine (vertebral, spinal cord), oncology (neoplasm, tumor, cancer, primary, secondary), tool, index, measure, frailty, and sarcopenia. The comprehensive search strategy included subject headings (MeSH terms) specific to the respective databases, along with keywords (full search strategy: Supplemental Content B).^24^ Pre-defined inclusion and exclusion criteria were used to screen abstracts (Table 1).

**Table 1.** Inclusion and Exclusion Criteria.

	Inclusion	Exclusion
Population	• Primary or secondary (metastatic) tumor of the spinal column or spinal cord	• Non-oncological populations (e.g. trauma, degenerative, congenital)
		• Patients who did not undergo spinal surgery for a spinal column or spinal cord tumor
Intervention	• Index (initial) surgery (minimally invasive or open)	• Non-operative management, including patients treated solely with radiotherapy or stereotactic radiosurgery, chemotherapy, or immunotherapy
		• Patients treated with kyphoplasty, vertebroplasty, radioablation, or biopsies
Study design (including tools and measures)	• English language	• Non-English language studies
	• Published after Jan 1^st^, 2000	• Conference abstracts without a published full text article
	• Full text articles	• Letters
	• Uses or applies a clinical frailty or sarcopenia tool (or measure) with a stated methodological design that can be completed with information collected from a patient history and/or physical examination, and/or radiographic and/or laboratory investigations	• Editorials
	• Human studies	• Absent description or reference to methodological design of frailty or sarcopenia measure
	• Study designs including randomized controlled trials, case-series, cohort studies (retrospective, prospective, ambispective)	
Outcomes	• Mortality (any time point)	
	• Intra-operative or post-operative morbidities (major or minor)	
	• Length of stay	
	• Hospital readmission	
	• Reoperation rate(s)	
	• Discharge disposition (including non routine discharge)	
	• Health related quality of life	
	• Functional outcomes (general)	
	• Cognitive assessment (any type)	
	• Frailty or sarcopenia trajectory	

### Study Selection

Study selection was performed using a two-step screening process. Covidence systematic review software was used to screen all results from the database searches. Upon upload of RIS files from each database, duplicate articles were automatically excluded by the software. A single reviewer (MAM) ensured accuracy of the duplicate removal algorithm, and additional duplicates were identified during screening. Institution and database of published studies were used to avoid inclusion of duplicate patient data. Two reviewers (MAM, MG) independently screened titles and abstracts using the pre-specified inclusion criteria. Selected full texts were assessed for eligibility and screened in duplicate. Cases of disagreement were resolved via open discussion.

### Data Collection

A standardized data extraction form was reviewed and refined by all authors prior to use (Supplemental Digital Content C). Study characteristics, frailty tools, and sarcopenia measures employed are detailed in Table 2 and Supplemental Digital Content D. Other variables extracted were relevant to frailty tools or sarcopenia measures and included component domains, individual items, operational definition(s) of frailty, cut off values, number of items per tool, setting and use of application, special training required for use, sarcopenia measurement technique(s), and prevalence of frailty or sarcopenia among the study cohort (Tables 3 and 4). Individual items were categorized as either modifiable or non-modifiable, based on whether components can or cannot be adjusted to raise or lower the risk of frailty, respectively (Supplemental Content D).^12^ Post-operative clinical outcomes recorded included mortality, adverse events (AEs), length of hospital stay, hospital re-admission, re-operation, non-routine discharge (NRD) (Tables 5 and 6). Follow-up rates were recorded.

**Table 2.** Study Demographics.

Study Author	Year	Journal	Study Design (Pro-, Retro-, Ambi-spective)	Sample Size	% Male	Database *(NIS; Nationwide Inpatient Sample, ACS-NSQIP; American College of Surgeons National Quality Improvement Program)*	Age Criteria (Years)	Mean or Median Age (Years)	Age Range (IQR or SD; years)	Study Population: Primary, Metastatic, or Both	Tumor Types	Frailty or Sarcopenia Tools Examined
Ahmed	2017	World neurosurg	Retrospective	1589	51.8% (n = 823)	NIS and ACS-NSQIP	None	47	28-61	Primary	Histologies not specified; benign tumors (55%), malignant tumors (45%)	PSTFI
Bakhsheshian	2022	TSJ	Retrospective	7772	52.20%	Health care cost and utilization project nationwide database	None	62.3	SD 12	Metastatic	Lung (12.2%), breast, (11.7%), prostate (15.4%), kidney, (8.8%), thyroid (2.8%), sarcoma (1.1%), lymphoma/myeloma (3.1%), melanoma (2.6%), GI/digestive organs (4.3%), unspecified (38.0%)	JHACG
Bongers	2022	TSJ	Retrospective	196	63% (n = 123)	Institutional database	≥18	62	53-70	Metastatic	Renal cell (14%), lung (13%), breast combined (9.7%), multiple myeloma (7.1%), colon and rectal (6.1%), hepatocellular (5.1%), sarcoma (4.6%), other (4.6%), NSCLC (4.1%), prostate combined (7.7%), thyroid (3.6%), melanoma (3.1%), unknown (3.1%), head and neck (2.6%), esophageal (2.6%), lymphoma (2.6%), gallbladder (2.6%), other urological (1.5%), pancreatic (1.0%)	L4 total muscle CSA (cm^2^)/height squared (m^2^); L4-VATA; L4-SATA
Bourassa-moreau	2020	TSJ	Retrospective	108	52.8% (n = 57)	Institutional database	None	62.5	35-84	Metastatic	Breast (22%), lung (17%), kidney (16%), prostate (12%), other (33.3%)	MFI-11; MSTFI; l3-TPA/VBA
Brinkmann	2021	JSO	Retrospective	48	52.1% (n = 25)	Institutional database	None	54	SD 13	Both	Sarcomas (64.6%, which includes 20 patients (41.7%) with chordoma), carcinoma (35%, which includes 15 patients (31.3%) with colorectal carcinoma)	PLVI
Charest-morin	2019	JNS spine	Ambispective	113	59% (n = 67)	Institutional database	None	49	SD 19	Both	Metastatic tumors: 13%, primary tumors: 87%; proportion of primary tumors: Malignant (66%) and benign (34%); primary bone tumor types: Chordoma (34%), chondrosarcoma (12%), GCT (8%), osteoblastoma/osteoid osteoma (8%), high-grade sarcoma (7%), MPNST (5%), hemangioma (4%), enchondroma/chondroma (4%), osteosarcoma (3%), others (7%); metastatic tumor types: Renal (33%), colon (27%), lung (20%), thyroid (13%)	mFI-11
De la Garza Ramos	2016	World neurosurg	Retrospective	4583	57.8% (n = 2650)	NIS and ACS-NSQIP	≥18	62	54-70	Metastatic	Lung (34.1%), breast (21.1%), prostate (21.1%), kidney (19.9%), thyroid (3.8%); other tumor histologies were excluded	MSTFI
De la Garza Ramos	2021	J clin neurosci	Retrospective	105	58.1% (n = 61)	Institutional database	None	61	SD 13	Metastatic	Hematologic (23.8%), prostate (16.2%), breast (14.3%), lung (13.3%), colorectal (6.7%), liver (5.7%), kidney (4.8%), other (15.2%)	MSTFI
Ehresman	2021	JNS spine	Retrospective	350	53.1% (n = 185)	Institutional database	≥18	57	SD 13.6	Both	Histologies specified only for tumor types with n = 20 or greater: Chordoma (14.9%), sarcoma (11.7%), lung primary (10.6%), plasma cell (9.1%), breast primary (8.9%), prostate primary (8.6%), renal primary (5.7%)	mFI-5
Elsamadicy	2022	JNS spine	Retrospective	11 480	Low frailty: 57.8% (n = 4095), intermediate frailty: 65.6% (n = 2729), high frailty: 74.5% (n = 175)	Health care cost and utilization Project's nationwide database	≥18	Low frailty: 61.8, intermediate frailty: 64.6, high frailty: 68.7	Low frailty: SD 11.2, intermediate frailty: SD 11.5, high frailty: SD 11.0	Metastatic	Histologies not specified	HFRS
Elsamadicy	2022	World neurosurg	Retrospective	1613	MFI-5 = 0: 56.1% (n = 393), MFI-5 >=1: 60.2% (n = 550), MSTFI = 0: 42.5% (n = 159), MSTFI >=1: 63.3% (n = 784)	ACS-NSQIP	≥18	MFI-5 = 0: 59, MFI-5 ≥ 1: 66; MSTFI = 0: 62, MSTFI ≥1: 64	MFI-5 = 0: 51-67, MFI-5 >=1: 59-73, MSTFI = 0: 53-69, MSTFI >=1: 56-71	Metastatic	Histologies not specified	mFI-5 and MSTFI
Elsamadicy	2022	GSJ	Retrospective	5955	Non-frail: 37.4% (n = 1756), frail: 31.7% (n = 400)	Health care cost and utilization nationwide database	≥18	Non-frail: 55.7, frail: 63.3	Non-frail: SD 16.4, frail: SD 16.2	Primary	Histologies not specified	HFRS
Gakhar	2015	ESJ	Ambispective	86	51.2% (n = 44)	Institutional database	None	Alive at 1 year: 62, dead at 1 year: 68	Alive at 1 year: 53-72, dead at 1 year: 62-76	Metastatic	*Alive at 1 year:* Lymphoma (31%), breast (23%), prostate (12%), renal (17%), lung (6%), other (6%), GI (6%); *Dead at 1 year:* Lung (28%), prostate (18%), renal (15%), other (15%), GI (12%), breast (6%), lymphoma (6%)	L3-TPA/VBA and L3-TPA/Height^2^
Hersh	2022	JNS spine	Retrospective	322	56.9% (n = 197)	Institutional database	≥18	59.5	SD 12	Metastatic	Lung (15%), breast (13%), prostate (11%), and renal (10%)	mFI-5 and MSTFI
Hu	2022	Clin nutrition	Retrospective	180	61.1% (n = 110)	Institutional database	≥18	58.3	22-85	Metastatic	Lung (39%), other (25%), liver (18%), breast (11%), hematopoietic (5%), kidney (2%)	L3-TPA/VBA and L3-TPA/Height^2^
Kazim	2022	Neurospine	Retrospective	4662	53% (n = 2470)	ACS-NSQIP	≥18	59	47-68	Both	Histologies not specified; primary tumors (37.6%), secondary (43.3%), unknown (19%)	mFI-5
Lakomkin	2018	Spine	Retrospective	2170	54% (n = 1171)	ACS-NSQIP	None	57	SD 16	Both	Histologies not specified; primary tumors (35.4%), secondary (42.9%), unknown (21.7%)	mFI-11
Massaad	2021	Neurosurgical focus	Retrospective	479	58.7% (n = 281)	Institutional database	≥18	64	55-71	Metastatic	Lung (18.8%), kidney (10.7%), breast (9.8%), blood (9.2%), prostate (8.8%), colon/rectal (5.9%), bone sarcoma (5.0%), thyroid (4.8%), head and neck (4.6%), skin (4.4%), other (17.9%)	MSTFI
Massaad	2021	JNS spine	Retrospective	88	73.9% (n = 65)	Institutional database	≥18	60.8	SD 11	Metastatic	Renal (100%)	mFI-11 and L3-SMI
Massaad	2022	JNS spine	Retrospective	303	57.8% (n = 175)	Institutional database	≥18	62	SD 12	Metastatic	GU (25%), lung (20%), GI (14%), breast (11%), hematology (7%), skin (5%), bone sarcoma (4%), head/neck (4%), thyroid (3%), soft-tissue sarcoma (3%), other (3%)	MSTFI, L3-SMI, SMD, VAT, SAT
Rothi	2019	J clin neurosci	Retrospective	41	63.4% (n = 27)	Institutional database	≥18	64	SD 9	Metastatic	Prostate (27%), unknown (15%), breast (15%), lungs (12%), renal (10%), bladder (5%), cervix (3%), thyroid (3%)	mFI-11
Zakaria	2020	Neurosurgery	Retrospective	271	Psoas tertile 1: 58.8% (n = 53), psoas tertile 2: 57.7% (n = 52), psoas tertile 3: 57.1% (n = 52)	Institutional database	None	Psoas tertile 1: 61.3, psoas tertile 2: 60.5, psoas tertile 3: 57.4	Psoas tertile 1: SD13, psoas tertile 2: SD 9, psoas tertile 3: SD 12	Metastatic	Lung (23%), prostate (15%), kidney (14%), breast (13%), hematopoietic (8%), GI (7%), nasopharynx (6%), thyroid (4%), liver (2%), skin (2%), other (7%)	L3-4 TPA

**Table 3.** Tools for Assessing Frailty in Spinal Oncology.

Frailty Measure	Study Author	Frailty Tool Component Domains	Operational Definition of Frailty (e.g Cumulative Deficit Definition, Phenotypic or Weighted Frailty Models); Specify Whether Scale is Dichotomous or Not, Specify if It has a Range of Points	Cutoff Values (e.g. Score 1-2, Pre Frail; Score 3-5 Frail, etc)	Number of Items of Tool	Setting of Use and Application	Special Tools or Training Required for Use	Prevalence of Frailty Among Cohort (Overall, %)	Prevalence of Frailty Among Cohort Stratified (by Score, %)
PSTFI	Ahmed 2017	Comorbidities; laboratory; nutrition; radiographic	Accumulation of deficits - dichotomous scale; range 0-9 points	No frailty (0); mild frailty (1); moderate frailty (2); severe frailty (3 or more)	9	Hospital	No	Any frailty (score ≥ 1) = 28.3%; no frailty = 71.7%	Score zero (71.7%), 1 (20.1%), 2 (6.0%), ≥3 (2.2%)
JHACG	Bakhsheshian 2022	Comorbidities, cognition, function, falls, socioeconomic, nutrition	Accumulation of deficits - dichotomous scale. Range: 0-1	Non-frail (0); frail (1 or more “diagnostic cluster” present)	10	Hospital	No	Any frailty (score ≥ 1) = 25.4%; no frailty = 74.6%	Frail group (25.4%); propensity matched to non-frail group (score 0) (25.4%)
mFI-11	Bourassa-Moreau 2020	Comorbidities, function, cognition	Accumulation of deficits - dichotomous scale. Range: 0-1	No frailty (0); pre-frail (mFI >zero and <.21), and frail (≥.21)	11	Hospital	No	Any frailty (score ≥1) = 81.5%; no frailty = 18.5%	Score zero = 18.5%; .09 = 49.1%; .18 = 17.6%; .27 = 8.3%; .36 = 4.6%; .45 = .9%
	Charest-Morin 2019		Accumulation of deficits - dichotomous scale. Range: 0-1	Frailty: ≥.21				NR	Mean mFI-11 score (total): .05 (.07); metastatic tumor group: .09 (.10); primary tumor group: .04 (.07); *P* = .0149 (primary vs metastatic)
	Lakomkin 2018		Accumulation of deficits - full range of mFI values used in order to avoid data loss as a result of dichotomization. Range: 0-1	No cut off values specified				NR	NR
	Massaad 2021 J neurosurg spine		Accumulation of deficits - continuous score. Range: 0-11	No frailty (0), mild frailty (1), moderate frailty (2), severe frailty ≥3				Any frailty (score ≥1) = 76.1%; no frailty = 23.9%	Score zero = 23.9%, 1 = 26.1%, 2 = 25%, ≥3 = 25%
	Rothi 2019 J clin neurosci		Accumulation of deficits - dichotomous scale. Range:0-1	Range zero to 1 (not frail to most frail)				NR	NR
MSTFI	De la Garza Ramos 2016	Comorbidity, surgical approach, laboratory, and nutrition	Accumulation of deficits - dichotomous scale. Range: 0-9	No frailty (0); mild frailty (1); moderate frailty (2); severe frailty (≥3); any frailty implied by scores ≥1	9	Hospital	No	Any frailty (score of 1 or more) (82.8%); no frailty (17.2%)	Score zero = 17.2%, 1 = 40.1%, 2 = 24.7%, 3 or more: 18%
	Bourassa-Moreau 2020							Any frailty (score 1 or more) = 74.1%, no frailty = 25.9%	Score zero = 15.7%, 1 = 30.6%, 2 = 19.4%, 3 = 24.1%
	De la Garza Ramos 2021							Any frailty (score of 1 or more) (91.4%); no frailty (8.6%)	Score zero = 8.6%, 1 = 33.3%, 2 = 37.1%, ≥3: 21.0%
	Elsamadicy 2022 world NSx							Any frailty (score of 1 or more) (76.8%); no frailty (23.2%)	Score zero = 23.2%, 1 = 36.2%, 2 = 26.2%, score 3 = 11.9%, score 4: 2.3%; ≥5 = .2%
	Hersh 2022 J neurosurg spine							Any frailty (score of 1 or more) (80.6%); no frailty (19.4%)	Score zero = 19.4%, 1 = 29.2%, 2 = 32.4%, score 3 = 14.2%, score 4: 3.8%; ≥5 = 1.2%
	Massaad 2021 neurosurg focus							Any frailty (score of 1 or more) (93.5%); no frailty (6.5%)	Score zero = 6.5%, 1 = 23.2%, 2 = 33.8%, ≥3 = 36.5%
	Massaad 2022 JNS spine							Any frailty (score of 1 or more) (95%); no frailty (5%)	Score zero = 5%, 1 = 25.4%, 2 = 31.0%, ≥3 = 38.6%
mFI-5	Ehresman 2021	Comorbidities, function	Accumulation of deficits - dichotomous scale. Range: 0-5	Cutoff values not specified within manuscript; mean mFI-5 score (0-5) reported	5	Hospital	No	NR	Mean mFI-5 score for the cohort: 1.1 ± 1.0
	Elsamadicy 2022 world NSx			No frailty (0); any frailty: ≥1				Any frailty (56.6%); No frailty (43.4%)	Score zero = 43.4%, 1 = 38.8%, 2 = 15.4%, 3 = 2.1%, 4 = .2%, 5 = 0%
	Hersh 2022 J neurosurg spine			Cutoff values not specified. Scores range from zero (not frail) to 5 (maximum frailty), implying any frailty as score of any score ≥1				Any frailty (score ≥1) (72.8%); no frailty (27.2%)	Score zero = 27.2%, 1 = 41.0%, 2 = 23.7%, 3 = 7.5%, 4 = 7.5%, 5 = .6%
	Kazim 2022			Least frail (0) to 5 (most frail); score of 1 (pre-frail), 2 (frail), and 3 or more (severely frail)				Any frailty (score ≥ 1) (49.6%); no frailty (50.4%)	Score zero = 50.4%, 1 = 34.8%, 2 = 13.2%, ≥3 = 1.9%
HFRS	Elsamadicy 2022 J neurosurg spine	Comorbidities, cognition, falls, function, continency, and visual-audio impairment, laboratory, nutrition and weight, mood, social support, other	Weighted instrument - dichotomous scale with weighted score	Low frailty (HFRS < 5), intermediate frailty (HFRS 5-15), and high frailty (HFRS > 15)	109	Hospital and community	Yes	All (100%) of patients have some degree of frailty	Low frailty (HFRS < 5): 61.7%, intermediate frailty (HFRS 5-15): 36.2%, high frailty (HFRS >15): 20.4%
	Elsamadicy 2022 global spine J			Non-frail (HFRS < 5) and frail (HFRS ≥ 5)				Frail: 21.2%, non-frail: 78.8%	Mean HFRS score, non-frail: 1.5 ± 1.4; frail: 8.3 ± 3.0

**Table 4.** Tools for Measuring Sarcopenia in Spinal Oncology.

Sarcopenia Measure	Study Author	Sarcopenia Calculation	Cutoff Values (e.g. Score 1-2, Pre Frail; Score 3-5 Frail, etc)	Prevalence of Sarcopenia Among Cohort	Setting of Use and Application	Special Measurement Technique, Tools, or Training Required for Use
L4 CSMA/Height^2^	Bongers 2022	Measurements using CT abdominal imaging at the level of the L4 vertebral body, including the CSA (sum of paraspinal and abdominal skeletal muscles)	<52.4 cm^2^/m^2^ for men, <38.5 cm^2^/m^2^ for women	Sarcopenic (42%); not sarcopenic (58%)	Hospital	Yes (measurements are performed using an in-house custom automated algorithm for segmentation analysis and requires inspection and adjustment of segmentation manually by a trained observer using horos DICOM viewer version 6.5.2, www.horosproject.com)
L3-4 TPA	Zakaria 2020	Measurements performed using CT abdominal imaging at the level of the L3-4 disc space or L4 pedicle; total cross sectional area of both psoas muscles	1st, 2nd, and 3rd tertiles	1st, 2nd, and 3rd tertiles for men and women, respectively; male (mean) L3-4 TPA ± SD, with minimum and maximum (range): 8.59 cm^2^ ± 1.31, 5.45-10.47 cm2 for tertile 1 (T1); 11.99 cm^2^ ± .80, 10.50-13.36 cm2 for tertile 2 (T2); and 15.50 cm^2^ ± 1.87, 13.37-21.08 cm2 for tertile 3 (T3); female (mean) L3-4 TPA ± SD, with minimum and maximum (range): 6.13 cm^2^ ± .80, 4.20-7.22 cm^2^ for T1; 8.04 cm^2^ ± .51, 7.24-9.05 cm2 for T2; and 10.68 cm^2^ ± 1.61, 9.06- 14.95 cm^2^ for T3	Hospital	Yes, free-hand region-of-interest tool, which calculates cross-sectional area
L3-TPA/VBA	Bourassa Moreau 2020	Using the axial abdominal CT image, *total* psoas muscle area was calculated and divided by the vertebral body area, both at the mid-L3 spinal level	1st, 2nd, 3rd, 4th quartiles	Median for cohort (no units provided): .93 (.49-2.62); per quartile: 1st quartile: .73 (.49-.81), 2nd quartile: .87 (.82-.93), 3rd quartile: 1.08 (.93-1.24), 4th quartile 1.53 (1.25-2.62)	Hospital	Yes; semi-automated volumetric calculation based on manual segmentation using PACS system
	Gakhar 2015			Alive at 1 year (arbitrary units): Median, 2.26 (IQR 1.70-2.67); per quartile: 1st quartile: 17%; 2nd quartile: 25%, 3rd quartile: 27%, 4th quartile: 31%; dead at 1 year: Median 1.95 (1.54- 2.29); per quartile: 1st quartile: 35%; 2nd quartile: 26%, 3rd quartile: 24%, 4th quartile: 15%		Yes; image J software (NIH, Maryland, USA) was used to measure and quantify vertebral and psoas muscle cross-sectional area
	Hu 2022		1st, 2nd, and 3rd tertiles	Male patients: .515 and .625 (cm2/cm2), respectively; female patients: .397 and .505 (cm^2^/cm^2^), respectively		Yes; image analysis software required, however, specific software not specified by author in the manuscript text (reference to prior work provided)
L3-TPA/Height^2^	Gakhar 2015	Using the axial abdominal CT image, *total* psoas muscle area was calculated and divided by patient height (squared)	1st, 2nd, 3rd, 4th quartiles	Correlation between L3-TPA/Height^2^ and L3-TPA/VBA is provided (*r* = .77, *P* < .0001), however, prevalence rates per quartile are not provided for L3-TPA/height (squared) (see Gakhar 2015, L3-TPA/VBA, for prevalence rates per quartile)	Hospital	Yes; image J software (NIH, Maryland, USA) was used to measure and quantify psoas muscle cross-sectional area
	Hu 2022		1st, 2nd, and 3rd tertiles	Male patients: .0248 and .0316 (cm^2^/cm^2^), respectively; female patients: .0182 and .0224 (cm^2^/cm^2^), respectively		Yes; image analysis software required, however, specific software not specified by author in the manuscript text (reference to prior work provided
L4-PLVI	Brinkmann 2021	Measurements performed using CT abdominal imaging at the level of L4 immediately inferior to the origin of the posterior elements; psoas:vertebral index is computed as the ratio between the *average* of the mean cross-sectional areas of the psoas muscles (right and left), divided by the L4 vertebral body cross sectional area	High (>.97) vs low (<.97), where .97 cuttoff represents the 50th percentile (cohort median)	50% of pts were sarcopenic (PLVI <.97; 50th percentile cutoff)	Hospital	Yes; segmentation software required, e.g. PACS system
L3-SMI (L3-CSMA/height squared)	Massaad J neurosurg spine 2021	Abdominal CT, L3 level, unit threshold range of −29 to 150, includes psoas, erector spinae, quadratus lumborum, transversus abdominis, external and internal oblique, and rectus abdominis muscles. The L3-SMI was calculated by measuring the CSMA (cm^2^) at this level divided by the patient height (m^2^)	Sarcopenia defined by L3-SMI <41 cm^2^/m^2^ in females, <43 cm^2^/m^2^ in males with BMI <25 kg/m^2^ and 53 cm^2^/m^2^ in males with BMI >25 kg/m^2^	Sarcopenic (51.1%); not sarcopenic (48.9%)	Hospital	Yes; segmentation software required; osirix version 8.0.1 (pixmeo SARL)
	Massaad 2022 JNS spine		Sarcopenia: <39 cm^2^/m^2^ for women and <55 cm^2^/m^2^ for men	Sarcopenic (67%); not sarcopenic (33%); L3-SMI: No sarcopenia, 53.1 (10.05) vs sarcopenia, 40.76 (7.20), *P* < .001		Yes (image retrieval using the medical imaging informatics bench to bedside (mi2b2) workbench and automated segmentation using the previously described, validated machine learning-based body composition analysis pipeline (MLBCAP) based on two convolutional neural networks that had been trained on abdominal CT data from examinations of 604 patients with pancreatic adenocarcinoma enrolled in a multi-institutional research protocol with source code available only); “DenseNet” for L3 CT slice selection and “U-net” for segmentation of skeletal muscle compartment
Body composition based clutering (composite of L3-SMI, SMD, VAT, SAT)	Massaad 2022 JNS spine	Abdo CT, L3 level, body composition profiles generated using k-means unsupervised clustering analysis to identify two groups of pts with similar patterns of L3-SMI (L3-CSMA/height squared), SMD (average radiation attenuation of the muscle tissue in HU), VAT, and SAT; before clustering SMI, SMD, SAT, and VAT measures were standardized to the same scale (mean zero ±1) to avoid possibility of any variable with more significant influence on cluster assignment; grouping based on only the findings of the body composition analysis and did not include clinical characteristics or subsequent outcomes	Clustering of patients on the basis of body composition analysis yielded an optimal number of two clusters; the optimal number of groups was determined using a combination cohesion/separation approach (i.e. silhouette index)	Cluster 1 (41%); cluster 2 (59%); cluster 1 vs cluster 2: SMI (cm^2^/m^2^) 50.13 (10.45) vs 41.16 (7.99), *P* < .001; SMD (HU), 31.13 (9.07) vs 35.67 (9.94), *P* < .001; SAT, 289.83 (109.31) vs 147.62 (57.80), *P* < .001; VAT, 239.26 (98.40) vs 82.28 (48.96), *P* < .001	Hospital	Yes; mi2b2 and MLBCAP (as above) with “DenseNet” for L3 CT slice selection and “U-net” for segmentation of the skeletal muscle, VAT, and SAT compartments; followed by L3-SMI calculation and calculation of the average attenuation of skeletal muscle measurement in HU
L3-SMD	Massaad 2022 JNS spine	Abdominal CT, L3 level, the average radiation attenuation for the skeletal muscle is measured in HU	Not cutoff values have been previously described or reported	SMD: No sarcopenia group, 36.42 (9.57) vs sarcopenia group, 32.53 (9.73), *P* = .001 (where sarcopenia was defined using L3-SMI cutoff of <39 cm^2^/m^2^ for women and <55 cm^2^/m^2^ for men); cluster 1, 31.13 (9.07) vs 35.67 (9.94), *P* < .001	Hospital	Yes; mi2b2 and MLBCAP (as above) with “DenseNet” for L3 CT slice selection and “U-net” for segmentation of skeletal muscle compartment, followed by measurement of SMD (average skeletal muscle radiation attenuation in HU)
L3-VAT	Massaad 2022 JNS spine	Abdominal CT, L3 level, cross-sectional area of the VAT is measured in cm^2^	Not cutoff values have been previously described or reported	VAT: No sarcopenia group, 165.54 (112.06) vs sarcopenia group, 137.15 (102.58), *P* = .029 (where sarcopenia was defined using L3-SMI cutoff of <39 cm^2^/m^2^ for women and <55 cm^2^/m^2^ for men); cluster 1, 239.26 (98.40) vs 82.28 (48.96), *P* < .001	Hospital	Yes; mi2b2 and MLBCAP (as above) with “DenseNet” for L3 CT slice selection and “U-net” for segmentation of visceral adipose compartment
L4-VAT	Bongers 2022	Abdominal CT, L4 level, VAT attenuation measured in HU	Not cutoff values have been previously described or reported	Median attenuation for cohort provided: -83 (IQR -90; -60) HU	Hospital	Yes (measurements are performed using an in-house custom automated algorithm for segmentation analysis and requires inspection and adjustment of segmentation manually by a trained observer using horos DICOM viewer version 6.5.2, www.horosproject.com)
L3-SAT	Massaad 2022 JNS spine	Abdominal CT, L3 level, cross-sectional area of the SAT is measured in cm^2^	Not cutoff values have been previously described or reported	SAT: No sarcopenia group, 239.64 (113.12) vs sarcopenia group, 189.16 (102.15), *P* < .001 (where sarcopenia was defined using L3-SMI cutoff of <39 cm^2^/m^2^ for women and <55 cm^2^/m^2^ for men); cluster 1, 289.83 (109.31) vs 147.62 (57.80), *P* < .001	Hospital	Yes; mi2b2 and MLBCAP (as above) with “DenseNet” (L3 CT slice selection) and “U-net” (segmentation of subcutaneous adipose compartment)
L4-SAT	Bongers 2022	Abdominal CT, L4 level, SAT attenuation measured in HU	Not cutoff values have been previously described or reported	Median attenuation for cohort provided: -94 (IQR -102;-85) HU	Hospital	Yes (measurements are performed using an in-house custom automated algorithm for segmentation analysis and requires inspection and adjustment of segmentation manually by a trained observer using horos DICOM viewer version 6.5.2, www.horosproject.com)

**Table 5.** Cllinical Outcomes Frailty.

Frailty Measure	Study Author	Clinical Outcomes Examined	Summary of Primary Study Finding Related to Frailty	Mortality	AEs (Peri-op)	Postop AEs (Any, Major, or Minor)	LOS (Days Unless Otherwise Specified)	Radmission Rates	Reoperation Rates	Non Routine Discharge	F/U Rate
PSTFI	Ahmed 2017	Postop AE; hospital LOS	Severely frail patients experienced increased complications but the PSTFI lacks predictive validity as the AUC for the ROC was low (.6042); mild and moderate frailty did not predict complications after external validation; hospital LOS was increased for frail patients compared to non-frail patients but this was not tested in the external validation analysis	NR	NR	Overall proportion of patients developing ≥1 major complications: 10.6% (6%, benign; 16.2%, malignant). Complication rates: Pleurisy, pneumothorax, or pulmonary collapse (5.8%); acute respiratory distress syndrome (3.7%); reintubation (1.4%); pneumonia (1.4%). Others had an incidence of <.1%. For patients with no frailty, mild frailty, moderate frailty, and severe frailty, complication rates were 5.7%, 18.8%, 29.2%, and 44.1% (*P* < .001). Internal validation cohort (univariate logistic regression): Mild (OR 3.83, 95CI 2.63-5.58); moderate (OR 6.80, 95CI 4.10-11.3); and severe (OR 13.05, 95CI 6.34-26.87) frailty were associated with developing complications (all *P* < .001). ROC AUC .6892. Subgroup analysis for benign tumors: Mild (OR 4.56; 95CI 2.28-9.11), moderate (OR 19.99; 95CI, 8.81-45.33); and severe (OR 47.15, 95CI 9.86, 225.50) frailty had higher odds of developing major complications (all *P* < .001); subgroup analysis for malignant tumors: Mild (OR 2.85; 95CI 1.80-4.50), moderate (OR 2.92, 95CI 1.51-6.45), severe (OR 6.02, 95CI, 2.63-13.75); all *P* < .001). External validation cohort (univariate regression): Any 30d postoperative complications: Mild frailty (OR 1.74, 95% CI .73-4.12, *P* = .207), moderate frailty (OR 2.36, 95% CI .59-9.40, *P* = .221), and severely frail (OR 9.4, 95% CI 1.41-63.3, *P* = .020). ROC AUC .6042	Median 5d (IQR, 3-9 days); increasing preoperative frailty index scores were associated with an increase in postoperative LOS. Patients without frailty had a mean LOS of 6.4 days ±.2 compared with 9.8 days ±.6, 14.4 days ±1.7, and 18.3 days ±2.6 for patients with mild, moderate, and severe frailty (all *P* < .05)	NR	NR	NR	NR
JHACG	Bakhsheshian 2022	Mortality, medical AEs, surgical AEs, hospital LOS, discharge disposition, care-related costs	Frailty was not associated with increased mortality; however, frail patients had increased AEs, hospital LOS, nonroutine discharge rates, and care costs compared to patients without frailty; the AUC was below the acceptable limit for AEs, and borderline acceptable for LOS; the AUC for mortality was only acceptable for pts undergoing lumbar procedures, not cervical or thoracic	No difference in mortality between frail (5.4%) and non-frail (4.8%) patients (OR 1.28, 95% CI (.88-1.86), *P* value = .19); AUCs, cervical (.613), thoracic (.573), lumbar (.750)	Intra-operative complications grouped with post-operative complications	Medical complications: Frail (62.2%) vs non-frail (50.5%) (OR 1.58, (1.33-1.86; *P* < .0001)); and surgical complications including peri-operative complications: Frail (18.2%) vs non-frail (12.5%) (OR 1.46, 95% (1.15-1.85; *P* = .0016)); AUC .675	Frail (19.9 d) vs non-frail (13.6 d) (OR = 2.65; 95% CI 2.09−3.37; *P* < .0001)); AUC .752	NR	NR	Frail (82.1%) vs non-frail (72.3%) (OR = 1.79; 95% CI 1.46−2.20; *P* < .0001)	NR
mFI-11	Bourassa Moreau 2020	Post-op AEs, 1 m and 3 m mortality	Higher mFI-11 scores were not associated with AEs and 1 m or 3 m mortality	Mean mFI-11 scores were not associated with 1 m or 3 m mortality	NR	287 AEs (92 patients (85%)) with a mean of 2.6 AEs per patient. Most common post-operative AEs were urinary tract infection (41%), electrolyte imbalance (27%), and delirium (23%); additional AE rates are not provided; mean mFI scores were not associated with presence of AEs; the bivariate correlation studies between predictive tools and number of postop AEs were significant for mFI, but mFI did not predict the number of postop AEs in multivariate logistic regression analysis	NR	NR	NR	NR	NR
	Charest-Morin 2019	Any AEs; in-hospital mortality; prolonged postop LOS; postoperative unplanned reoperation, ICU admission	mFI -11scores were not associated with development of any AEs, ICU admission, or reoperation; increased mFI-11 was reported to predict LOS but the 95% CI crosses 1.0	No in-hospital deaths occurred in the cohort	Prevalence of ≥1 intraop AE (overall): 34%; prevalence of intra-op AEs: Massive blood loss (>2 L/3h or > 5 L/24h) (20%), dural tear (16%); neurological injury (7%), vascular injury (3%), hardware malposition (3%), airway/ventilation (3%), others (5%); see post-op AE (right) for comparison of mFI (no AE vs any AE)	Prevalence of ≥1 postop AE (overall): 76%; prevalence by type of postop AE: anemia (34%), UTI (24%), delirium (17%), wound healing complication (17%), pneumonia (16%), severe neuropathic pain (16%), respiratory failure (12%), wound infection (11%), dysphagia (10%); electrolytes disturbance (8%), constipation (7%), thromboembolic event (6%), persistent CSF leak (5%), dysphonia (4%), sepsis (4%); others (21%); wound complications were seen in 28% (from wound drainage [17%] to deep wound infection [11%] requiring further procedures). The median number of AEs per patient was 2 (IQR 0-4); thirty-two patients (28%) required an ICU stay; mFI-11: No ICU group, .048 (.075) vs ICU group, .051 (.065); *P* = .85; mean mFI scores: No AE, .036 (.07) vs any AE, .05 (.07), *P* = 0.3	Median LOS 16d (IQR 9-32); high mFI predicted increased LOS on uni- and multivariate analyses: .72, 95% CI .09-1.35, *P* = .025; note the 95% CI crosses 1.0)	NR	mFI did not predict increased odds of reoperation in uni- or multivariate analyses: OR, 40.19 (95% CI .13-129046; *P* = .90)	NR	NR
	Lakomkin 2018	30 d mortality, major or minor AEs, prolonged LOS	mFI-11 scores were associated with increased odds of 30 d mortality and prolonged LOS but not major or minor AEs	30d mortality rate: 4.5%; mFI scores were significantly associated with 30d mortality (OR = 15.6, 95% CI: 1.31-184.96, *P* = .03; AUC .860); when stratifying the analysis according to primary and secondary tumors, mFI did not predict mortality for primary tumors. mFI predicted 30d mortality for secondary tumors (OR 63.80, 95% CI 2.80-1451.69; *P* = .009; AUC, .827)	NR	Major AEs: 11.5%; minor AEs: 19.8%; mFI scores were not significantly associated with major or minor AEs (OR not provided). When stratifying the analysis according to primary and secondary tumors, mFI did not predict major or minor AEs for either	Prolonged LOS: 25.7%; mFI scores were significantly associated with prolonged LOS (OR = 14.9, 95% CI: 3.45-64.47, *P* < .001; AUC .749); when stratifying the analysis according to primary and secondary tumors, mFI did predict prolonged LOS for primary tumors (OR 323.60, 95% CI 16.78-6240.95; *P* < .001; AUC .784). mFI did not predict prolonged LOS for secondary tumors	NR	NR	NR	NR
	Massaad 2021 J neurosurg spine	OS	Increased mFI-11 scores were not associated with poor OS	Mortality during followup period (median 17 m, range 1-104 months): 64.7%; in univariate analysis, mFI scores were not associated with OS: Score 1, HR 1.0 (95% CI .48-2.2), *P* = .93; score 2, HR 1.5 (95% CI, .69-3.1), *P* = .33; score ≥3, HR 1.9 (.92 (95% CI .92-4.0), *P* = .08	NR	NR	NR	NR	NR	NR	Median followup: 17m (range 1-104); 64.7% had died during this period
	Rothi 2019 J clin neurosci	OS	mFI-11 was not correlated with survival	38 deceased by the end of the study period with a 30-day mortality of 14.6% and a 1-year mortality of 73.2%; the mFI was found to have poor correlation with survivorship (r = -.089, *P* = .595)	NR	AEs: GI (17%), ongoing pain (15%), decline in functional status (10%), respiratory (5%), incisional (5%)	NR	NR	NR	NR	Mean followup was 7.7 months; no rate provided
MSTFI	De la Garza Ramos 2016	Peri-op major AEs; hospital LOS; in-hospital mortality (internal validation sample), 30 d mortality (external validation sample)	Frailty was associated with mortality with internal validation (NIS dataset); there was no significant association between frailty (any severity) and mortality during external validation (ACS-NSQIP dataset); frailty (any severity) was associated with development of one or more major peri-operative complications only during internal validation (NIS dataset); mild frailty and moderate frailty were not associated with one or more major peri-operative complications during external validation using the ACS-NSQIP dataset; only severe frailty maintained significance as a predictor of development of one or more complications with external validation; note, low poor predictive function (AUC .672)	In hospital death rate: 3.0%; internal validation model for in-hospital mortality: Mortality rate was 1.0%, 1.6%, 4.8%, 5.5%, 5.3%, 9.6%, .0%,and .0% for patients with 0, 1, 2, 3, 4, 5, 6, and 7 points on the index score, respectively; compared with patients with no frailty, patients with moderate frailty (OR 5.15; 95% CI 2.44-10.86) and severe frailty (OR 5.74; 95% CI 2.69-12.24) had significantly increased odds of mortality (all *P* < .001); external validation model for 30d mortality: Overall mortality among 610 separate patients was 12.1%; mortality rates were 7.3%, 12.2%, 13.0%, and 24.2% for patients with no frailty, mild frailty, moderate frailty, and severe frailty, respectively (*P* = .094); the ROC AUC for the mortality model was .565	Overall peri-op complication rate: 882 patients (19.3%) who developed at least 1 perioperative complication, with the most common being pleurisy/pneumothorax/pulmonary collapse in 7.9% of patients; internal validation model for peri-op complications: Compared with patients with no frailty, patients with mild frailty (OR 1.88; 95% CI 1.33-2.66), moderate frailty (OR 3.83; 95% CI 2.71-5.41), and severe frailty (OR 6.97; 95% CI 4.98-9.74) had significantly increased odds of developing a major complication (all *P* < .001). The complication rate was 6.7%, 12.0%, 21.7%, 36.4%, 45.5%, 69.2%, 85.7%, and 100.0% for patients with 0, 1, 2, 3, 4, 5, 6, and 7 comorbidities, respectively. External validation model for peri-op major complications: .9%, 4.8%, 3.7%, and 24.4% for patients with no frailty (reference), mild frailty (OR 5.47, 95% CI .71-41.93, *P* = .102), moderate frailty (OR 4.18, 95% CI .49-35.22, *P* = .188), and severe frailty (OR 34.36 (4.02-293.49, *P* = .001), respectively; the ROC AUC for the complication model was .672	Grouped with peri-operative complications	Average LOS: 10.5 (SD 8.6) days for all patients; LOS was significantly different between patients with no frailty and mild frailty (3.3, SD .4 days, *P* < .001), moderate frailty (5.6, SD .4 days, *P* < .001), and severe frailty (6.4, SD .4 days, *P* < .001)	NR	NR	NR	NR
	Bourassa moreau 2020	Post-op AEs, mortality (1 m and 3 m)	Higher MSTFI scores were associated with 3 m mortality, but not AEs or 1 m mortality	Mean MSTFI scores were no different for 1 m mortality; however, higher MSTFI was associated with 3 m mortality (2.04 (SD .99) vs 1.47 (SD 1.00), *P* = .012)	NR	Overall, AE rates are provided above (see above entry for mFI); mean MSTFI scores were no different between patients with or without AEs; the bivariate correlation studies between predictive tools and number of postop AEs were not significant for MSTFI.	NR	NR	NR	NR	NR
	De la Garza Ramos 2021	Peri-op or post-op AEs (30 d or 90 d)	MSTFI did not have predictive accuracy based on the pre-defined clinical utility threshold of AUC .70 in the ROC analysis for any complications at 30 d post-op (.62 (.52 – .73)), major complications at 30 d post-op (.64 (.52 – .76)), or any complications at 90 d post-op (.55 (.44-.67)), respectively	NR	30d complications: Any morbidity (36.2%), major complications (21.9%), DVT (13.3%), wound infection (8.6%), pneumonia (6%), ARDS (2.9%), reintubation (2.9%), PE (1.9%), UTI (1.9%), C diff (1.9%), sepsis (1.9%), AKI (1.9%), epidural abscess with meningitis (.9%), epidural hematoma (.9%), cardiac arrhythmia (.9%)	90d complications: ≥1 AE (71.4%); major complications rate per MSTFI score: No difference across scores, zero (11.1%), 1 (11.4%), 2 (28.2%), 3 (31.8%); *P* = .170; all complications rate per MSTFI score: No difference across scores, zero (22.2%), 1 (22.9%), 2 (46.2%), 3 (45.5%); *P* = .114	NR	NR	NR	NR	NR
	Elsamadicy 2022 world NSx	Any postop AEs; hospital LOS; non routine discharge; unplanned readmission	In uni- and multivariate analyses, increased MSTFI scores predicted hospital LOS, non routine discharge, any AE, and unplanned readmission	NR	“Any AEs” reported, see “postop AEs” (right)	Increased MSTFI predicted development of any AEs in univariate (OR 1.46 (1.29-1.65), *P* < .001) and multivariate (aOR, 1.43 (1.24-1.64), *P* < .001) analyses. Prevalence of any AEs: MSTFI score = 0, 8.6% vs Score 1 ≥ 20.3%)	Increased MSTFI predicted prolong hospital LOS in univariate (RR 1.51 (.78-2.24), *P* < .001) and multivariate (aRR, 1.14 (.48-1.81), *P* = .001) analyses. Mean LOS (days): MSTFI score = 0, 6.2 ± 12.6 vs Score ≥1, 8.6 ± 16.4	Increased MSTFI predicted unplanned readmission in univariate (OR 1.33 (1.17 - 1.51), *P* < .001) and multivariate (aOR, 1.22 (1.05-1.43), *P* = .011) analyses; prevalence of unplanned readmission: MSTFI score 0, 8.6% vs Score ≥1: 16.5%; prevalence of readmission related to principal surgery: MSTFI score 0, 6.7% vs MSTFI ≥1, 9.9%	MSTFI ability to predict of reoperation was not analyzed in uni- or multivariate analyses. Prevalence of reoperation: MSTFI score 0, 4.8% vs Score ≥1, 6.7%)	Increased MSTFI predicted non routine discharge in univariate (OR 1.58 (1.43-1.75), *P* < .001) and multivariate (aOR, 1.56 (1.38-1.76), *P* < .001) analyses. Prevalence of nonroutine discharge: MSTFI score 0, 29.1%, score ≥1: 47.8%)	NR
	Hersh 2022	Any postop AEs; prolonged hospital LOS; non routine discharge	In uni- and multivariate analyses, increased MSTFI scores predicted development of any AE and prolonged hospital LOS	NR	NR	24.0% experienced ≥1 postop AE. Increased MSTFI predicted development of any postop AEs in univariate (OR 1.36 (95% CI 1.17-1.59, *P* < .01) and multivariate (OR per point, 1.41 (95% CI 1.12-1.77, *P* < .01)	Mean ± SD LOS was 11.2 ± 9.9 days. Increased MSTFI predicted prolonged hospital LOS in univariate (OR 1.57 (95% CI 1.26-1.95, *P* < .01) and multivariate (OR per point, 1.63 (95% CI 1.29-2.05, *P* < .01)	NR	NR	44.5% of patients had nonroutine discharge. Increased MSTFI is reported as predicting nonroutine discharge in univariate (OR 1.14 (95% CI .96-1.34, *P* < .01) and multivariate (OR per point, 1.69 (95% CI 1.36-2.11, *P* < .01) analyses. However, it is noted that confidence interval for the OR in the univariate analysis crosses 1.0	100% of patients included had at least 30d followup unless they died before 30d postop
	Massaad 2021 neurosurg focus	Major AEs within 30 d postop; prolonged LOS; in-hospital mortality	Frailty score was not associated with increased risk of 30 d in-hospital mortality, 30 d postop major AEs, or prolonged LOS. The MSTFI demonstrated poor discrimination for predicting complications and in-hospital mortality	Inpatient mortality: 1.9%. Patients with severe frailty did not have an increased risk of in-hospital death compared with patients with moderate frailty (RR 6.46, 95% CI .81-52.09) and mild frailty (RR 4.44, 95% CI .55-35.6). The AUROC was .69 (95% CI .54-.85) for predicting in-hospital mortality demonstrating that the MSTFI performed fairly across severity risk groups. The calibration slope (.58, *P* = .06) was less than 1, indicating the MSTFI was overestimating the predicted risk of in-hospital mortality for high-risk patients (severe frailty), while underestimating for patients at low risk	NR	Patients with severe frailty had the highest rate of complications after spine surgery (49/136, 36.1%) compared with patients with less severe frailty, however, mild frailty (RR, 1.05, 95% CI .51-2.20, NS), moderate frailty (RR 1.28, 95% CI .64-2.57, NS), and severe frailty (RR 1.48, 95% CI .74-2.93, NS) were not associated with increased risk of developing major AEs within 30d postop. The MSTFI demonstrated poor discrimination for 30d postop AEs (AUROC, .56, 95% CI .50-.62. The MSTFI was poorly calibrated (calibration intercept .23, *P* < .001; calibration slope .25, *P* = .09) for the predicted and observed probabilities of 30d postop AEs	Hospital LOS: 7.8 d. Frailty was not associated with LOS (no frailty: 6.2d; mild frailty: 7.3d; moderate frailty, 7.9d; and severe frailty, 8.9d	NR	NR	NR	NR
	Massaad 2022 J neurosurg spine	12 m mortality; although any major AEs and LOS are reported, frailty is not analyzed as a predictive variable for those outcomes	In multivariate analysis for cluster 1 (clustered according to SMI scores), there was no difference in 12 m mortality between no frailty, mild frailty and moderate frailty groups, whereas patients with severe frailty had increased odds of mortality compared to mild frailty; for cluster 2 (clustered according to body composition analysis scores), the association with mortality was the same as for model 1 (i.e. only severe frailty had increased odds of mortality compared to mild frailty); frailty was not analyzed as a predictor of AEs or LOS	NR	NR	NR	NR	NR	NR	NR	NR
mFI-5	Ehresman	Hospital LOS and non-routine discharge	Greater frailty (higher mFI-5 scores) predicted increased odds of non-routine discharge in multivariate analysis; higher mFI-5 scores were not associated with prolonged LOS	NR	NR	NR	Higher mFI-5 scores did not predict increased hospital LOS (1.1 ± 1.0, LOS <10 days vs 1.1 ± 1.0, LOS ≥10 days, *P* = .53)	NR	NR	Higher mFI-5 scores were associated with non-routine discharge (home, .8 ± .9 vs subacute rehabilitation/skilled nursing facility, 1.5 ± 1.0 vs acute care inpatient rehabilitation, 1.4 ± 1.0; *P* < .001); higher mFI-5 score (OR 1.90 per point, 95% CI 1.35-2.69, *P* < .001) predicted increased odds of non-routine discharge in both univariate and multivariate analysis	Follow-up rate not specified but outcomes corresponded to index admission only
	Elsamadicy 2022 world NSx	Any postop AEs; hospital LOS; non routine discharge; unplanned readmission	In uni- and multivariate analysis, increased mFI-5 scores predicted nonroutine discharge and development of any AEs but did not predict unplanned readmission or prolong hospital LOS. In multivariate analysis, increased mFI-5 scores predicted nonroutine discharge and presence of any AEs only	NR	“Any AEs” reported, see “postop AEs” (right)	Increased mFI-5 predicted development of any AEs in univariate (OR 1.21 (1.03-1.41), *P* = .017) and multivariate (aOR, 1.23 (1.02-1.49), *P* = .032) analyses. Prevalence of any AEs: mFI-5 score = 0, 16.3% vs Score 1 ≥ 18.5%)	Increased mFI-5 did not predict prolong hospital LOS in univariate (RR .50 (-.46-1.46), *P* = .308) or multivariate (aRR, .50 (-.40-1.39), *P* = .276) analyses. Mean LOS (days): mFI-5 score = 0, 7.6 ± 16.3 vs Score ≥1, 8.4 ± 15.1	mFI-5 did not predict unplanned readmission in univariate (OR 1.05 (.89 - 1.25), *P* = .571) or multivariate (aOR, .94 (.76-1.16), *P* = .576) analyses; prevalence of unplanned readmission: mFI-5 score 0, 14.6% vs Score ≥1: 14.7%; prevalence of readmission related to principal surgery: mFI-5 score 0, 9.0% vs mFI-5 ≥ 1, 9.3%	mFI-5 ability to predict reoperation was not analyzed in uni- or multivariate analyses. Prevalence of reoperation: mFI-5 score 0, 5.1% vs Score ≥1, 7.1%)	Increased mFI-5 predicted non routine discharge in univariate (OR 1.55 (1.36-1.76), *P* < .001) and multivariate (aOR, 1.37 (1.17-1.60), *P* < .001) analyses. Prevalence of nonroutine discharge: mFI-5 score 0, 35.4%, score ≥1: 49.7%)	NR
	Hersh 2022	Any postop AEs; prolonged hospital LOS; non routine discharge	In uni- and multivariate analyses, increased mFI-5 scores predicted development of any AE, prolonged hospital LOS, and nonroutine discharge	NR	NR	24.0% experienced ≥1 postoperative complication. Increased mFI-5 predicted development of any postop AEs in univariate (OR 1.47 (95% CI 1.14-1.90, *P* < .01) and multivariate (OR per point, 1.95 (95% CI 1.23-3.09, *P* < .01)	Mean ± SD LOS was 11.2 ± 9.9 days. Increased mFI-5 predicted prolonged hospital LOS in univariate (OR 1.38 (95% CI 1.07-1.78, *P* < .01) and multivariate (OR per point, 1.67 (95% CI 1.06-2.63, *P* < .01)	NR	NR	44.5% of patients had nonroutine discharge. Increased mFI-5 is reported as predicting nonroutine discharge in univariate (OR 1.84 (95% CI 1.45-2.35, *P* < .01) and multivariate (OR per point, 2.65 (95% CI 1.74-4.04, *P* < .01) analyses	100% of patients included had at least 30d followup unless they died before 30d postop
	Kazim	Postoperative AEs (major); 30 d mortality; prolonged hospital LOS; nonroutine discharge; unplanned readmission or reoperation	In uni- and multivariate analyses, frailty was associated with increased mortality, major AEs, unplanned readmission, prolonged hospital LOS, and non routine discharge. Severe frailty was associated with mortality, major AEs, reoperation, hospital LOS, and non routine discharge. mFI-5 discrimination for mortality was fair (AUC .743)	Postop 30d mortality: 1.6%; highest incidence of mortality was among the “severely frail” group: 13.5%. Univariate analysis for association between frailty and 30d mortality: Prefrail: OR, 1.38 (95% CI .71-2.7); frail: OR, 5.73 (95% CI 3.12-10.52), severely frail: OR 20.2 (95% CI 9.40-43.3); notably, the prefrail univariate result is marked as statistically significant, however, the confidence crosses 1.0; multivariate analysis for association between frailty and 30d mortality: Prefrail: OR 1.31 (95% CI .85-2.15); frail: OR, 4.01 (95% CI 3.34 - 9.12); severely frail: OR 16.4 (95% CI 11.21-35.44); similarly, the prefrail multivariate result is marked as statistically significant, however, the confidence crosses 1.0; ROC for mFI-5 and 30d mortality (AUC = .743; 95% CI .661-.825, *P* < .001)	NR	Postop major AEs rate: 10.6%; postop minor AE rate: 15.6%. Most common AEs was blood transfusion (12.6%), UTI (3.5%), and pneumonia (2.3%). Highest incidence of major AE was among the “severely frail” group: 23.6%. Univariate analysis for association between frailty and major AEs: Prefrail: OR, 1.58 (95% CI .92-2.72); frail: OR, 2.14 (95% CI 1.27-3.61), severely frail: OR 3.09 (95% CI 1.86-5.16); notably, the prefrail univariate result is marked as statistically significant, however, the confidence crosses 1.0; multivariate analysis for association between frailty and major AEs: Prefrail: OR 1.62 (95% CI .98-2.23); frail: OR, 2.20 (95% CI 1.43 - 3.32); severely frail: OR 3.02 (95% CI 1.97-4.56); similarly, the prefrail multivariate result is marked as statistically significant, however, the confidence crosses 1.0	Median hospital LOS was 5d (IQR, 3-9); univariate analysis for association between frailty and hospital LOS: Prefrail: OR, 1.24 (95% CI 1.09-1.67); frail: OR, 1.89 (95% CI 1.32-2.13), severely frail: OR 3.01 (95% CI 1.01-4.53); multivariate analysis for association between frailty and major AEs: Prefrail: OR 1.21 (95% CI 1.01-1.39); frail: OR, 1.91 (95% CI 1.42 - 2.19); severely frail: OR 2.94 (95% CI 2.32-4.21)	Readmission rate: 9.3%; univariate analysis for association between frailty and unplanned readmission: Prefrail: OR, 1.29 (95% CI 1.03-1.62); frail: OR, 1.77 (95% CI 1.33-2.34), severely frail: OR 1.78 (95% CI .92-3.40); the severely frail group OR for unplanned readmission is marked as statistically significant but the CI crosses 1.0; multivariate analysis for association between frailty and unplanned readmission: Prefrail: OR 1.23 (95% CI 1.01-1.59); frail: OR, 1.74 (95% CI 1.21 - 2.04); severely frail: OR 1.76 (95% CI .88-3.7); the severely frail group OR for unplanned readmission is marked as statistically significant but the CI crosses 1.0	Reoperation rate: 5.1%; univariate analysis for association between frailty and reoperation: Prefrail: OR, 1.39 (95% CI 1.04 - 1.84); frail: OR, 1.10 (95% CI .73-1.68), severely frail: OR 2.13 (95% CI 1.01-4.53); the severely frail group OR for reoperation is marked as statistically significant but the CI includes 1.0; multivariate analysis for association between frailty and reoperation: Prefrail: OR, 1.32 (95% CI 1.01 - 1.82); frail: OR, 1.06 (95% CI .83-1.54), severely frail: OR 2.07 (95% CI 1.00-4.17)	Nonroutine discharge rate: 34.8%; univariate analysis for association between frailty and nonroutine discharge: Prefrail: OR, 1.56 (95% CI 1.36-1.79); frail: OR, 2.52 (95% CI 2.10-3.02), severely frail: OR 2.00 (95% CI 1.30-3.07); multivariate analysis for association between frailty and major AEs: Prefrail: OR 1.52 (95% CI 1.33-1.75); frail: OR, 2.48 (95% CI 2.15 - 2.98); severely frail: OR 1.91 (95% CI 1.25-2.88)	30 days; the authors state there is no loss to follow-up in the NSQIP database
HFRS	Elsamadicy 2022 JNS spine	Intra-op AEs including transfusion of blood products; postoperative AEs (any); hospital LOS; discharge disposition	Higher frailty was associated with higher likelihood of experiencing any postop AEs; in multivariate analyses, higher frailty was associated with longer LOS, higher rates of nonroutine discharges	NR	The proportion of patients in each cohort who required blood (low frailty: 12.8%; intermediate frailty: 19.8%; high frailty: 36.2%; *P* < .001) or platelet transfusions (low frailty: 2.5%; intermediate frailty: 4.9%; high frailty: 8.5%; *P* = .002) increased along with worsening frailty	The proportion of patients in each cohort who experienced any postop AE significantly increased along with increasing frailty (low frailty: 29.2%; intermediate frailty: 53.8%; high frailty: 76.6%; *P* < .001). Postop AE rates per frailty category (low, intermediate, or high frailty): UTI (low: 1.0%; intermediate: 18.0%; high: 59.6%; *P* < .001), acute posthemorrhagic anemia (low: 22.8%; intermediate: 30.6%; high: 36.2% cord *P* < .001), pressure ulcer (low:1.4%; intermediate: 6.4%; high: 14.9%; *P* < .001), and acute DVT (low: 3.2%; intermediate: 5.8%; high: 17.0%; *P* < .001). Other AEs associated with frailty: PE (*P* = .041), postprocedural shock (*P* = .018), and wound disruption (*P* < .001)	Mean LOS (low frailty: 7.9 ± 5.0 days; intermediate frailty: 14.4 ± 13.4 days; high frailty: 24.1 ± 18.6 days; *P* < .001) increased with increasing frailty; intermediate and high frailty were each found to be significant predictors of both prolonged LOS (intermediate: OR 3.75 [95% CI 2.96-4.75], *P* < .001; high: OR 7.33 [95% CI 3.47-15.51]; *P* < .001)	NR	NR	Rate of nonroutine discharge (low frailty: 40.4%; intermediate frailty: 60.6%; high frailty: 70.2%; *P* < .001) increased with increasing frailty; intermediate and high frailty were each found to be significant predictors of nonroutine discharge (intermediate: OR 2.05 [95% CI 1.68-2.51], *P* < .001; high: OR 5.06 [95% CI 1.93-13.30], *P* = .001)	NR
	Elsamadicy 2022 GSJ	Periop and postop AEs; hospital LOS; nonroutine discharge	Frailty (HFRS ≥ 5) was associated with longer hospital LOS and higher rate of non-routine discharge in univariate and multivariate analyses. Frailty was not associated with rate of intraop CSF leak, dural tear, or intraop blood transfusion. The ability of frailty to predict postop AEs was not analyzed in uni- or multivariate analyses	NR	There was no difference in CSF leak/dural tears (non-frail: 1.3% vs frail: 2.0%, *P* = .402) or blood transfusions (non-frail: 1.2% vs frail: 2.8%, *P* = .065)	The frail cohort experienced more postoperative complications (non-frail: 8.7% vs Frail: 39.3%, *P* < .001); UTI (non-frail: 1.1% vs frail: 24.2%, *P* < .001), pressure ulcer (non-frail: .2% vs frail: 4.4%, *P* < .001), acute post-hemorrhagic anemia (non-frail: 4.7% vs frail: 8.7%, *P* = .017), post-procedural shock (non-frail: .0% vs frail: .8%, *P* = .007), and acute DVT (non-frail: .6% vs frail: 2.4%,*P* = .013); there was no difference in rates of post-procedural fever, wound disruption, or nervous system complications (all *P* > .05)	Mean LOS (non-frail: 4.9 ± 3.7 vs Frail: 9.2 ± 6.7, *P* < .001); frailty was found to be a significant independent predictor of prolonged LOS in univariate (OR 6.55 (CI 4.77-9.00), *P* < .001) and multivariate (OR 6.24 (CI 4.27 - 9.10), *P* < .001) analyses	NR	NR	Frail patients had higher rates of non-routine discharge (non-frail: 31.0% vs frail: 66.7%, *P* < .001). Frailty was found to be a significant independent predictor of nonroutine discharge in univariate (OR 4.55 (CI 3.44-6.02)) and multivariate (OR 2.99 (CI 2.14 - 4.18), *P* < .001) analyses, *P* < .001	NR

**Table 6.** Clinical Outcomes Sarcopenia.

Sarcopenia Measure	Study Author	Clinical Outcomes Examined	Summary of Primary Study Finding Related to Frailty or Sarcopenia	Mortality	Intraop AEs Including OR Time or RBC Transfusions	Postop AEs (Any, Major, or Minor)	Length of Stay (Days Unless Otherwise Specified)	Radmission Rates	Reoperation Rates	Non Routine Discharge	Follow-Up Rate
L4 CSMA/Height^2^	Bongers 2022	Mortality (90 d and 1 y)	At 90-days, sarcopenia was not associated with mortality after adjusting for clinical factors. At 1-year, presence of sarcopenia was associated with an increased risk of mortality after adjusting for clinical factors, including modified bauer score	Overall mortality for the cohort: 24% (90 d), 54% (1 y), respectively; bivariate analysis for 90 d mortality: L4 CSMA/height2 (HR, 2.29; 95% CI, 1.26-4.19; *P* < .01) was associated with mortality; multivariate analysis for 90 d mortality: L4 CSMA/height2 (HR, 1.46; 85%CI, .72-2.96; *P* = .29) not associated with mortality; bivariate analysis for 1 year mortality: L4 CSMA/height2 (HR, 2.00; 95% CI, 1.35-2.98; *P* < .01) was associated with mortality; multivariate analysis for 1y mortality: Sarcopenia (66%) vs 41% (no sarcopenia) (HR, 1.68; 95% confidence interval, 1.08−2.61; *P* = .02) after adjusting for various clinical factors including primary tumor type, ECOG performance status, additional metastases, neurology status, and systemic therapy. This association held after separately adjusting for modified bauer score (HR, 1.58; 95% CI, 1.04-2.40; *P* = .03); no difference in mortality was observed between the CT group and (excluded) non-CT group (*P* > .05 for 90d and 1 y, respectively)	NR	NR	NR	NR	NR	NR	90 d (97.4%); 1 y (86.9%)
L3-4 TPA	Zakaria 2022	Peri-op AEs, 30 d, 90 d mortality	Smallest L3-4 TPA (T1) was not associated with higher 30 d mortality after adjusting for covariates; however, T1 was associated with increased odds of both 90d and overall mortality compared to middle (T2) and largest (T3) L3-4 TPA in multivariate analyses, respectively; there was no association between T1 and postop AEs or postop improvement in preop neurological deficit in multivariate analysis, respectively	30 d mortality: T1 (smallest), 12.2%; T2 (middle), 8.8%; T3 (largest), 2.2%; T3 had lower 30d mortality vs T2 (*P* = .049) vs T1 (*P* = .009), respectively; univariate OR (95% CI): T2 vs T1, .55 [.40-.76] *P* < .001; T3 vs T1, .41 [.30-.56] *P* < .001; T3 vs T2, .73 [.53-1.00] *P* = .056; multivariate OR (95% CI): T2 vs T1, .87 [.26-2.94] *P* = .82; T3 vs T1, .27 [.04-1.98] *P* = .199; T3 vs T2, .29 [.05-1.66] *P* = .167; 90d mortality: T1, 44.4%; T2, 21.1%; T3, 13.1%; T2 had lower 90d mortality vs T1 (*P* = .001); T3 had lower 90d mortality vs T1 (*P* < .001), respectively; univariate OR (95% CI): T2 vs T1, .33 [.17-.64] *P* = .001; T3 vs T1, .18 [.09-.39] *P* < .001; T3 vs T2, .56 [.25-1.25] *P* = .160; multivariate OR (95% CI): T2 vs T1, .24 [.09-.62] *P* = .003; T3 vs T1, .16 [.05-.44] *P* < .001; T3 vs T2, .67 [.24-1.81] *P* = .434; overall mortality: Univariate OR (95% CI): T2 vs T1, .56 [.41-.77] *P* < .001; T3 vs T1, .41 [.30-.56] *P* < .001; T3 vs T2, .73 [.53-1.00] *P* = .056; multivariate OR (95% CI): T2 vs T1, .51 [.36-.74] *P* < .001; T3 vs T1, .46 [.31-.67] *P* < .001; T3 vs T2, .89 [.66-1.27] *P* = .529	NR	Postoperative new neurological deficit T1, 11.1%; T2, 11.1%; T3, 5.6%; *P* = .375; SSI: T1, 5.5%; T2, 3.3%; T3, 2.2%; *P* = .442; SSD: T1, 2.2%; T2, 1.1%; T3, 2.2%; *P* = 1.000; DVT: T1, 5.5%; T2, 4.4%; T3, 3.3%; *P* = .715; PE: T1, 3.3%; T2, 1.1%; T3, 4.4%; *P* = .542; MI: T1, 1.1%; T2, .0%; T3, .0%; *P* = .664; UTI: T1, 6.7%; T2, 1.1%; T3, 1.1%; *P* = .038; PNA: T1, 5.5%; T2, 2.2%; T3, 1.1%; *P* = .184; prolonged ICU stay (>3 d): T1, 10.0%; T2, 6.6%; T3, 13.3%; *P* = .329; CVA: T1, 2.2%; T2, 2.2%; T3, .0%; *P* = .401; postop improvement in preop neurological deficit: Univariate OR (95% CI): T2 vs T1, .65 [.32-1.31] *P* = .234; T3 vs T1, 1.11 [.52-2.33] *P* = .779; T3 vs T2, 1.70 [.83-3.46] *P* = .141; multivariate OR (95% CI): T2 vs T1, .47 [.17-1.28] *P* = .137; T3 vs T1, 1.94 [.57-6.58] *P* 0 = .290; T3 vs T2, 4.11 [1.27-13.3] *P* = .018; any postop AE: Univariate OR (95% CI): T2 vs T1, .50 [.27-.93] *P* = .030; T3 vs T1, .55 [.29-1.01] *P* = .057; T3 vs T2, 1.09 [.57-2.08] *P* = .778; multivariate OR (95% CI): T2 vs T1, .58 [.28-1.20] *P* = .143; T3 vs T1, .72 [.34-1.54] *P* = .407; T3 vs T2, 1.25 [.59-2.62] *P* = .548	NR	Unplanned readmission: T1 (smallest), 14.4%; T2 (middle), 13.3%; T3 (largest), 6.5%; no difference among tertiles (*P* = .199)	Unplanned reoperation: T1 (smallest), 7.7%; T2 (middle), 8.8%; T3 (largest), 5.4%; no difference among tertiles (*P* = .672)	NR	NR
L3-TPA/VBA	Bourassa Moreau 2020	Post-operative AEs, mortality (1 m and 3 m)	Lower L3-TPA/VBA scores was associated with increased post-op AEs, 1 m and 3 m mortality	Lower L3-TPA/VBA scores were associated with 1m mortality (.72 (SD .12) vs 1.08 (SD .40), *P* = .008) and 3m mortality (.86 (SD .27) vs 1.12 (SD .41), *P* < .001)	NR	287 AEs (92 patients (85%)) with a mean of 2.6 AEs per patient. Most common post-operative AEs were urinary tract infection (41%), electro-lyte imbalance (27%), and delirium (23%); additional AE rates are not provided, rather they are qualitatively presented in Figure 3 of manuscript; lower sarcopenia (L3-TPA/VBA) scores were associated with the occurrence of at least one post-operative AE (1.07 (SD .40) vs 1.25 (SD .52; *P* = .031); bivariate correlation studies between predictive tools and number of postop AEs were significant for L3-TPA/VBA; only L3-TPA/VBA predicted the number of postop AEs L3-TPA/VBA in multivariate logistic regression analysis and for every 1 unit increase in L3-TPA/VBA, odds of experiencing AE was reduced by .29 (95% CI .09-.93), *P* = .037)	NR	NR	NR	NR	NR
	Gakhar 2015	12 mortality	There was no difference in muscle mass between patients alive or dead at 12 months post-operative follow-up, however, 12 m mortality was increased among those in the lowest quartile compared to those in the highest quartile	Individuals who died within a year did not have lower lean muscle mass on initial CT compared to those alive at the end of 1 year (alive at 1 year: 2.26 (1.70-2.67) vs dead at 1 year: 1.95 (1.54-2.29), *P* = .05; pre-specified significance was for *P* values <.05). Calculated normalized lean muscle mass was analysed as quartiles, with 23.8% mortality in the highest quartile within a year compared to 57.1% mortality in the lowest quartile (*P* = .02). Individuals with a lung, GI, or ‘‘other’’ primary malignancies were more likely to be dead at 1 year, with all three groups having 1-year survival rates less than 50%. These groups are also the groups with the lowest muscle mass on CT at presentation. Of the malignancies recorded, lung primary patients had a significantly lower muscle mass on CT than breast and lymphoma patients (*P* < .01). There was no significant difference in muscle mass at presentation between all other malignancy groupings	NR	NR	NR	NR	NR	NR	12 m or time of death if earlier: 100%
	Hu 2022	Mortality (90 d, 1 y, overall)	In multivariate logistic regression, T3 L3-TPA/VBA was associated with decreased 90 d mortality compared to T1 or T2, respectively. Significance was maintained after adjusting for numerous preop variables known to influence postop mortality. However, no difference in 12 m mortality was observed between tertiles. T3 was associated with decreased overall mortality compared to only T1, however significance was not maintained after adjusting for preop demographic variables and neurological status, nor after adjusting for tumor type and systemic disease burden, which are known to influence postop mortality in this patient group	Actual 90 d mortality: T1: 36.7% (22 of 60) vs T3: 6.7% (4 of 60), *P* < .001; and T2: 35.0% (21 of 60) vs T3: 6.7% (4 of 60), *P* < .001). Actual 12 m mortality: T1: 73.3% (44 of 60) vs T3: 51.6% (31 of 60), *P* = .014. In multivariate logistic regression analysis, T3 L3-TPA/VBA was associated with decreased 90d mortality compared with either T1 (OR .24, 95% CI .09-.60) or T2 (OR .27, 95% CI .10-.69), respectively. These findings were maintained after adjusting for preop demographic variables and neurological score, as well as primary tumor type and systemic disease burden, respectively. However, no difference in 90d mortality was observed between patients in T1 and T2. L3-TPA/VBA was not associated with 12m mortality in multivariate regression (T1 vs 2, 2 vs 3. and 1 vs 3, all confidence intervals cross 1.0). T3 was associated with decreased overall survival in multivariate regression analysis compared to T1 (HR,0.62, 95% CI .41-.95). This difference was not maintained after adjusting for preop demographic variables and neurological score, nor after adjusting for primary tumor type and systemic disease burden, respectively. There was no difference between T3 and 2, nor between T1 and 2, regarding overall mortality	NR	NR	NR	NR	NR	NR	NR
L4-PLVI	Brinkmann 2021	OS, DFS, intraop AE, postop AEs, reoperation	Lower L4-PLVI (<.97 50th percentile, median cohort value) was not associated with OS, or disease free survival; further, there was no difference between lower or higher L4-PLVI for intraoperative complications, wound complications, ambulatory status, or re-operation rates postoperatively	OS at 2-, 5-, and 10-years were 82%, 62%, and 56%, respectively; PLVI <.97 was not associated with disease free survival (HR 2.04, 95% CI .68-6.12, *P* = .20) or OS (HR 1.57, 95% CI .60-4.06, *P* = .34)	PLVI <.97 vs > .97: No difference in RBC units transfused during the procedure (12 (SD 15) vs 10 (SD 18), *P* = .75) or operative time (1093, SD 502 mins vs 1048, SD 623 mins, *P* = .77)	PLVI <.97 vs > .97 for entire cohort: No difference in wound complications (n = 13, 54% vs n = 13, 54%, OR = 1.0, 95% CI .32-3.11, *P* = 1.0), deep infection (n = 8, 33% vs n = 9, 38%), OR = .83, 95% CI .25-2.72, *P* = 1.0); subgroup analysis of only patients with primary sarcoma (n = 31, 65%): PLVI <.97 was not associated with all cause complications (OR 1.66, 95% CI .33-8.42, *P* .69), deep wound infections (OR .6, 95% CI .11-3.03, *P* = .69), or wound complications (OR 1.45, 95% CI .34-6.11, *P* = .72)	NR	NR	PLVI <.97 vs > .97: No difference in reoperation (n = 13, 54% vs n = 10, 42%, OR = 1.65, 95% CI .52-5.18, *P* = .56)	NR	NR
L3-TPA/Height^2^	Gakhar 2015	12 m mortality	There was no difference in muscle mass between patients alive or dead at 12 months post-operative follow-up, however, 12 m mortality was increased among those in the lowest quartile compared to those in the highest quartile. L3-TPA/VBA correlates well with L3-TPA/Height^2^	Quartiles are not distinctly presented for L3-TPA/Height 2, however, they are presented for L3-TPA/VBA, which correlates well with L3-TPA/Height2 (r = .77, *P* < .0001)	NR	NR	NR	NR	NR	NR	12m or time of death if earlier: 100%
	Hu 2022	Mortality (90 d, 1 y, overall)	In multivariate logistic regression, T3 L3-TPA/Height2 was associated with decreased 90 d mortality compared to T1 or T2, respectively. However, no difference in 12 m mortality was observed between tertiles. T3 was associated with decreased overall mortality compared only to T1, however significance was not maintained after adjusting for preop demographic variables and neurological status which are known to influence postop mortality in this patient group	In multivariate logistic regression analysis, T3 L3-TPA/Height2 was associated with decreased 90d mortality compared with either T1 (OR .18, 95% CI .06-.46) or T2 (OR .26 (.09-.68). These findings were maintained after adjusting for preop demographic variables and neurological score, as well as primary tumor type and systemic disease burden, respectively. However, no difference in mortality was observed between patients in T1 and T2.; L3-TPA/Height2 was not associated with 12m mortality in multivariate regression (T1 vs 2, 2 vs 3. and 1 vs 3, all confidence intervals cross 1.0). T3 was associated with decreased overall survival in multivariate regression analysis compared to T1 (HR,0.66, 95% CI .44-.99). This difference was not maintained after adjusting for preop demographic variables and neurological score. There was no difference between T3 and 2, nor between T1 and 2, regarding overall mortality	NR	NR	NR	NR	NR	NR	NR
L3-SMI	Massaad 2021 J neurosurg spine	OS	L3-SMI was not associated with poor survival	Mortality during followup period (see right): 64.7%; in univariate analysis, L3-SMI was not associated with OS: Sarcopenia, HR .93 (95% CI .54-1.6), *P* = .78	NR	NR	NR	NR	NR	NR	Median followup: 17m (range 1-104); 64.7% had died during this period
	Massaad 2022 J neurosurg spine	Inpatient mortality, 90 d, 12 m mortality, OS, major AEs, LOS >7d	Lower L3-SMI was not associated with inpatient, 90 d, 12 m mortality, OS, major AEs, or LOS >7 d	Inpatient mortality: 1%; no sarcopenia (2%) vs sarcopenia (.5%), *P* = .52; OS (%): No sarcopenia (79%), sarcopenia (78.8%); No difference identified for 90d and 12m mortality; OS (m): No sarcopenia, 13m (8-22), sarcopenia, 7m (5-10); Kaplan-meier estimates of OS at 3m: No sarcopenia, 69.0% (60.4-78.7), sarcopenia (63.8% (57.4-70.8); at 6m: No sarcopenia, 60.8% (51.9-71.2), sarcopenia, 50.0% (43.5-57.5), at 12m: No sarcopenia, 49.7% (40.7-60.6), sarcopenia, 38.3% (32.1-45.7); uni- and multi-variate cox regression analyses for sarcopenia based on L3-SMI did not reveal a statistically significant increased risk of death at 12m (univariate: HR, 1.38 (95% CI .99-1.93, *P* = .06); multivariate: HR, 1.28 (95% CI .91-1.79, *P* = .15)	NR	Any AEs: 30%; major AEs: No sarcopenia (32.6%) vs sarcopenia (28.9%), *P* = .59; low L3-SMI was not associated with a higher rate of postop AEs	LOS (median): 6d (4-8), LOS >7 days (32.6%); LOS >7d: No sarcopenia (31%), sarcopenia (33.5%), *P* = .76	NR	NR	NR	NR
Body composition based clutering (composite of L3-SMI, SMD, VAT, SAT)	Massaad 2022 J neurosurg spine	Inpatient mortality, any AE, major AEs, LOS >7 d	Patients in cluster 2 (high-risk cluster) had an increased risk of mortality at 12 m postop, compared to cluster 1 (low-risk group); there was no difference between cluster 1 and 2 with respect to major AEs, LOS > 7 d, or inpatient mortality between cluster 1 and 2; cluster 2 had lower L3-SMI (mean ± SD 41.16 ± 7.99 vs 50.13 ± 10.45 cm^2^/m^2^), lower SAT (147.62± 57.80 vs 289.83 ± 109.31 cm^2^), lower VAT (82.28 ± 48.96 vs 239.26 ± 98.40 cm^2^), higher SMD (35.67 ± 9.94 vs 31.13± 9.07 hounsfield units (HU)) compared to cluster 1	Inpatient mortality: Cluster 1 (low risk) (1.6%) vs cluster 2 (high risk) (.6%), *P* = .74; overall mortality rate: Cluster 1 (71.0%), cluster 2 (80.4%); survival: Cluster 1, 15m (9-26), cluster 2, 6m (5-9); Kaplan-meier estimates of OS at 3m: Cluster 1, 68.4% (60.6-77.1), cluster 2, 63.5% (56.8-71.0); at 6m: Cluster 1, 62.5% (54.5-71.7), cluster 2, 47.5% (40.6-55.5), at 12m: Cluster 1, 51.7% (43.5-61.4), cluster 2, 35.5% (29.1-43.3); uni- and multi-variate cox regression analyses for sarcopenia based on cluster based body composition analyses did reveal a statistically significant increased risk of 12m mortality among cluster 2 (univariate: HR, 1.51 (95% CI 1.10-2.08, *P* = .01); multivariate: HR, 1.45 (95% CI 1.05-2.01, *P* = .02)	NR	Major AEs: Cluster 1 (32.2%) vs Cluster 2 (28.6%), *P* = .58	LOS > 7d: Cluster 1 (33.9%) vs Cluster 2 (31.8%), *P* = .80	NR	NR	NR	NR
L3-SMD	Massaad 2022 J neurosurg spine	Inpatient, 90 d, and 12 m mortality; major AEs, LOS > 7 d	Low L3-SMD was associated LOS > 7 d, but was not associated with inpatient, 90 d, or 12 m mortality, or major AEs	For the overall cohort, there was no difference in L3-SMD between those alive or deceased for inpatient, 90d, or 12m time points	NR	Low SMD was not associated with higher AEs; for the overall cohort, there was no difference in L3-SMD between those experiencing AEs and not experiencing AEs	Of 303 patients, 99 (32.6%) had LOS >7 days and were more likely to have low SMD (mean 30.87 vs 35.23 HU, 95% CI 1.98-6.73, *P* < .001)	NR	NR	NR	NR
L3-VAT	Massaad 2022 J neurosurg spine	Inpatient, 90 d, and 12 m mortality; major AEs, LOS >7 d	Low L3-VAT was not associated with inpatient, 90 d, or 12 m mortality, major AEs or LOS >7 d	For the overall cohort, there was no difference in L3-VAT between those alive or deceased for inpatient, 90d, or 12m time points	NR	Low VAT was not associated with higher AEs; for the overall cohort, there was no difference in L3-VAT between those experiencing AEs and not experiencing AEs	For the overall cohort, there was no difference in L3-VAT between those with LOS >7d or 7d or less	NR	NR	NR	NR
L4-VAT	Bongers 2022	Mortality (90 d and 1 y)	VAT attenuation was not associated with mortality at 90 d or 1 year, after adjusting for clinical factors	Overall mortality for the cohort: 24% (90d), 54% (1y), respectively; bivariate analysis for 90d mortality: L4-VAT (HR, 1.01; 95% CI, 1.00-1.03; *P* = .07) was not associated with mortality; bivariate analysis for 1 year mortality: L4-VAT (HR, 1.02; 95% CI, 1.01-1.03; *P* < .01) was associated with mortality; multivariate analysis for 1y mortality: VAT attenuation (HR, 1.01; 95% CI, .99-1.03; *P* = .53)	NR	NR	NR	NR	NR	NR	90d (97.4%); 1y (86.9%)
L3-SAT	Massaad 2022 J neurosurg spine	Inpatient, 90 d, and 12 m mortality; major AEs, LOS >7 d	Low L3-SAT was associated increased risk of 12 m mortality, but was not associated with inpatient or 90 d mortality, major AEs, or LOS >7 d	12 m mortality: Low SAT (190.30) vs high SAT (225.90 cm^2^/m^2^), 95% CI 10.89-60.30, *P* < .001); for the overall cohort, there was no difference in L3-SAT between those alive or deceased for inpatient, or 90d time points	NR	Low SAT was not associated with higher AEs; for the overall cohort, there was no difference in L3-SAT between those experiencing AEs and not experiencing AEs	For the overall cohort, there was no difference in L3-SAT between those with LOS >7d or 7d or less	NR	NR	NR	NR
L4-SAT	Bongers 2022	Mortality (90 d and 1 y)	SAT attenuation was not associated with mortality at 90 d or 1 year, after adjusting for clinical factors	Overall mortality for the cohort: 24% (90d), 54% (1y), respectively; bivariate analysis for 90d mortality: L4-SAT (HR, 1.02; 95% CI, 1.00-1.04; *P* = .04) was associated with mortality; multivariate analysis for 90d mortality: SAT attenuation (HR, .98; 95% CI, .96-1.01; *P* = .06); bivariate analysis for 1 year mortality: L4-SAT (HR, 1.02; 95% CI, 1.01-1.04; *P* < .01) was associated with mortality; multivariate analysis for 1y mortality: SAT attenuation (HR, .99; 95% CI, .98-1.02; *P* = .89)	NR	NR	NR	NR	NR	NR	90d (97.4%); 1y (86.9%)

### Clinimetric Assessment

Frailty and sarcopenia tools or measures included were evaluated for their clinimetric properties (MAM, RCM) (Tables 7 and 8). Definitions were adapted based on those previously reported by expert working groups (e.g., Consensus-based Standards for Health Measurement Instruments (COSMIN)),^12,28-31^ and prior application toward clinimetric assessment in the context of general spine surgery populations (for definitions: Table 7).^12^ Clinimetric properties included objectivity, feasibility, validity (content, construct, predictive), reliability, responsiveness, floor and/or ceiling effect(s), and current clinical application (e.g. risk stratification or frailty/sarcopenia trajectory). The patient population (primary, secondary, both) in which each tool may be applied with the highest degree of sensitivity was provided based on the full clinimetric assessment.

**Table 7.** Clinimetric Properties Frailty.

Frailty Measure	Study	Objective	Feasible	Content Validity	Construct Validity	Predictive Validity	Reliability	Responsiveness	Floor and Ceiling Effect	Current Clinical Application (e.g. Risk Stratification or Frailty Trajectory)	Sensitive Population
PSTFI	Ahmed 2017	+	+	−	NE	−	NE	NE	Floor	Risk stratification	Cannot determine
JHACG	Bakhsheshian 2022	−	+	−	?	?	NE	NE	Floor	Risk stratification	Cannot determine
mFI-11	Bourassa-Moreau 2020	+	+	?	?	−	NE	NE	Floor	Risk stratification	Cannot determine
	Charest-Morin 2019				NE	−	NE	NE	Floor	Risk stratification	Cannot determine
	Lakomkin 2018				−	?	NE	NE	?	Risk stratification	Cannot determine
	Massaad 2021 J neurosurg spine				−	−	NE	NE	Both	Risk stratification	Cannot determine
	Rothi 2019 J clin neurosci				−	−	NE	NE	?	Risk stratification	Cannot determine
MSTFI	De la Garza Ramos 2016	+	+	−	NE	−	NE	NE	Both	Risk stratification	Cannot determine
	Bourassa-Moreau 2020				−	?	NE	NE	Both	Risk stratification	Cannot determine
	De la Garza Ramos 2021				?	−	NE	NE	Ceiling	Risk stratification	Cannot determine
	Elsamadicy 2022 world NSx				?	+	NE	NE	Floor	Risk stratification	Metastatic disease
	Hersh 2022 J neurosurg spine				?	+	NE	NE	Floor	Risk stratification	Metastatic disease
	Massaad 2021 neurosurg focus				NE	−	NE	NE	Ceiling	Risk stratification	Cannot determine
	Massaad 2022 JNS spine				?	?	NE	NE	Ceiling	Risk stratification	Cannot determine
mFI-5	Ehresman 2021	+	+	−	−	?	NE	NE	Floor	Risk stratification	Cannot determine
	Elsamadicy 2022 world NSx				−	?	NE	NE	Floor	Risk stratification	Cannot determine
	Hersh 2022 J neurosurg spine				?	+	NE	NE	Floor	Risk stratification	Metastatic disease
	Kazim 2022				NE	+	NE	NE	Floor	Risk stratification	Primary and metastatic disease
HFRS	Elsamadicy 2022 J neurosurg spine	+	−	?	NE	+	NE	NE	Both	Risk stratification	Metastatic disease
	Elsamadicy 2022 global spine J				NE	+	NE	NE	Floor	Risk stratification	Cannot determine

Definitions:^12,32-36^ Objectivity: “item or subscale is not subjective to the bias, personal judgement, or cultural background of patients, their families or healthcare providers”; Feasibility: “item or subscale that is applicable or generalizable to spine oncology patient populations regardless of tumor type” and “easily obtainable from a standard spine history, physical examination, medical record, and routine laboratory tests, without the need for special equipment”; Construct validity: “scores on tool relate to other measures in a manner that is consistent with theoretically derived hypotheses […]” regarding frailty or sarcopenia, “assessed by testing predefined and specific hypotheses about expected correlations between known groups”, and measures may include the Spearman rank coefficient or Pearson correlation coefficient; content validity: items included in a tool should be “relevant, comprehensible, and represent a comprehensive sample with respect to the construct of interest and study population”, “methods for item selection should be reported/justified and piloted, with target population”; reliability: “degree to which patients can be distinguished from each other (despite measurement error)”, “time period for repeated administrations may be provided”, and intraclass coefficient (ICC) agreement or weighted Cohen’s Kappa coefficient (ICC or weight Kappa should be .70 or greater for sample of at least 50); responsiveness: “reflects ability of a tool to detect clinically important changes over time, even if changes are small”, is a measure of longitudinal validity; and “Should be assessed by testing predefined hypotheses about expected correlations between changes in measures (or expected differences in changes between known groups)”; floor and ceiling effects: “Present if more than 15% of respondents achieved the lowest (floor) or highest (ceiling) possible score”.

**Table 8.** Clinimetric Properties Sarcopenia.

Sarcopenia Measure	Study	Construct Validity	Validity Predictive	Reliability	Responsiveness	Current Clinical Application (e.g. Risk Stratification or Frailty Trajectory)	Sensitive Population
L4 CSMA/Height^2^	Bongers 2022	NE	?	NE	NE	Risk stratification	Cannot be determined
L3-4 TPA	Zakaria 2022	NE	?	NE	?	Risk stratification	Cannot be determined
L3-TPA/VBA	Bourassa Moreau 2020	?	+	+	NE	Risk stratification	Metastatic
	Gakhar 2015	?	?		NE	Risk stratification	Cannot be determined
	Hu 2022	?	?		NE	Risk stratification	Cannot be determined
L4-PLVI	Brinkmann 2021	NE	−	NE	NE	Risk stratification	Cannot be determined
L3-TPA/Height^2^	Gakhar 2015	?	?	+	NE	Risk stratification	Cannot be determined
	Hu 2022	?	?	+	NE	Risk stratification	Cannot be determined
L3-SMI	Massaad 2021 J neurosurg spine	−	−	NE	NE	Risk stratification	Cannot be determined
	Massaad 2022 J neurosurg spine	−	−	?	NE	Risk stratification	Cannot be determined
Body composition based clustering (composite of L3-SMI, SMD, VAT, SAT)	Massaad 2022 J neurosurg spine	−	?	?	NE	Risk stratification	Cannot be determined
L3-SMD	Massaad 2022 J neurosurg spine	?	−	?	NE	Risk stratification	Cannot be determined
L3-VAT	Massaad 2022 J neurosurg spine	?	−	?	NE	Risk stratification	Cannot be determined
L4-VATA	Bongers 2022	NE	−	NE	NE	Risk stratification	Cannot be determined
L3-SAT	Massaad 2022 J neurosurg spine	?	?	?	NE	Risk stratification	Cannot be determined
L4-SATA	Bongers 2022	NE	−	NE	NE	Risk stratification	Cannot be determined

### Study Bias and Evidence Appraisal

The Newcastle Ottawa Scale risk of bias assessment tool for observational studies was used to assess study bias (MAM, AC).^37^ Included studies were also appraised by two reviewers (MAM, MG) using the “Oxford Levels of Evidence 2” grading system.^38^ Disagreements were resolved via open, full-text review.

## Results

### Literature Search and Selection

**Figure 1.**
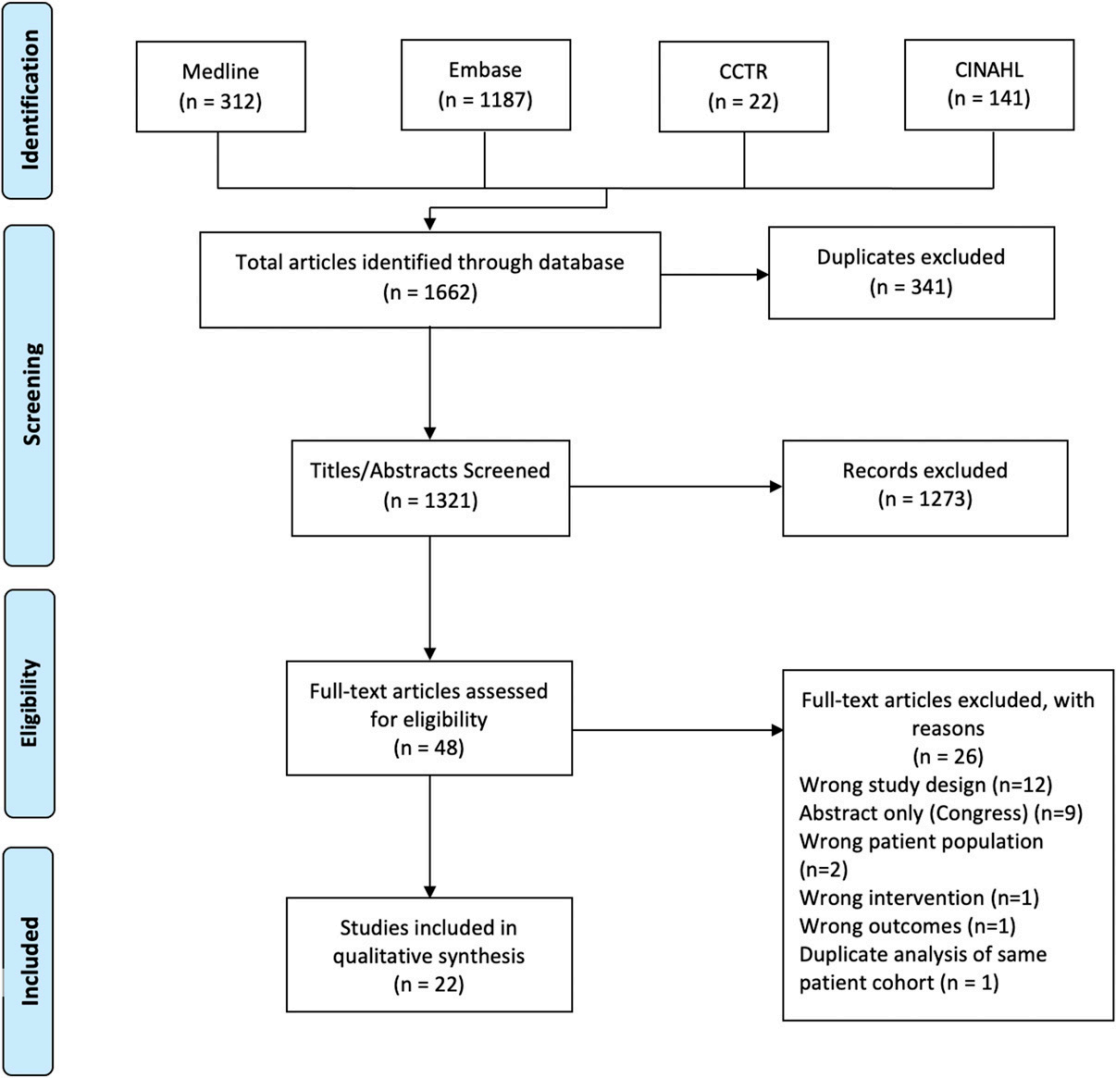
PRISMA flow diagram.

Our initial database search yielded 1662 articles (Figure 1). After duplicates were removed, 1321 records were screened at the title/abstract level and 1273 articles were excluded. Full texts were obtained for 48 studies to assess eligibility. Twenty-six articles were excluded via full-text assessment and twenty-two articles were included for qualitative synthesis.^8-11,17,18,22,39-53^

### Study and Cohort Characteristics

Study descriptive characteristics are presented in Table 2. Study designs were either retrospective (n = 20; 91%) or ambispective (n = 2; 9%). The total patient sample across all studies was 42 514, including 23 786 (66%) and 18 728 (44%) male and female patients, respectively. The average age was 59.7 years. Fourteen (63%) studies analyzed institutional databases, whereas 3 (14%) studies used data from the Health care Cost and Utilization Project National Inpatient Sample (NIS) database, 3 (14%) used the American College of Surgeons National Surgery Quality Improvement Program (AC-NSQIP) database, and 2 (9%) used a combination of the NIS and the ACS-NSQIP database. Tumor types were metastatic (n = 15 studies; 68%), primary (n = 2 studies; 9%), or both (n = 5 studies; 23%). The three most common tumor histologies were lung, breast, and prostate.

### Frailty Tools

Seventeen (77%) studies utilized frailty tools for risk stratification and outcome prognostication (Table 3). Tools included the PSTFI, JHACG, mFI-11, MSTFI, mFI-5, and HFRS. Components, domains, items, and their modifiability are described in Supplemental Content D. The MSTFI was the most employed frailty measurement tool (7 studies). The PSTFI and JHACG were each used in a single study, whereas the mFI-11, mFI-5, and HFRS were used in 5, 4, and 2 studies, respectively. Comorbidities was the most common component domain and was included in all tools (Table 3). Other common domains included function, cognition, nutritional status, and laboratory parameters. Frailty was most frequently operationalized according to an accumulation of deficits definition. Only the HFRS defines frailty using a weighted instrument. A single point score or more was categorized as either “pre-frail” or “frail” on the PSTFI, JHACG, and MTSFI. Cut-off values were either not reported or varied across studies examining the mFI-5 and mFI-11. The number of individual items per frailty tools ranged from 5-109. The overall mean prevalence of frailty across cohorts was 69%, and the range varied from 21%-100% (Table 3).

### Sarcopenia Measures

Eight (36%) studies used various measures for sarcopenia (Table 4). These included the L4-Cross Sectional Muscle Area (CSMA)/height^2^, L3-4 Total Psoas Area (TPA), L3-TPA/Vertebral Body Area (L3-TPA/VBA), L3-TPA/height^2^, L4-Psoas:Lumbar Vertebra Index (L4-PLVI), L3-Spinal Muscle Index (L3-SMI) (L3-CSMA/height^2^), BCA-based clustering (i.e. composite of L3-SMI, L3-Spinal Muscle Quality (i.e. radiodensity) (SMQ), L3-Visceral Adipose Tissue (L3-VAT), L3-Subcutaneous Adipose Tissue (L3-SAT)), L4-VAT, and L4-SAT. The most common sarcopenia measure used was L3-TPA/VBA (3 studies) followed by both L3-TPA/height^2^ (2 studies) and L3-SMI (L3-CSMA/height^2^; 2 studies). All sarcopenia measures were employed in the hospital setting and required special techniques or training for use. The overall prevalence of sarcopenia widely varied among the cohort with a range of 155-67%; while some studies reported overall prevalence, others reported prevalence stratified by quartiles (Table 4).

### Clinical Outcomes – Studies Employing Frailty Tools

Table 5 describes clinical outcomes, stratified by frailty tool. Nine studies examined mortality, 8 examined perioperative AEs, and 17 examined postoperative AEs. Furthermore, 14 studies examined hospital LOS, 3 examined readmission rates, 4 examined reoperation rates, and 9 examined NRD. The mFI-5 predicted mortality in a single study, the MSTFI in 1 of 3 studies, and the mFI-11 in 1 of 4 studies. The JHACG did not predict mortality in a single study examining it. The PSTFI and JHACG each predicted postoperative AEs in a single study. The mFI-5 predicted postoperative AEs in 3 of 3 studies, whereas the mFI-11 did not predict frailty in 3 of 3 studies. The MSTFI predicted postoperative AEs in 3 of 6 studies, and the HFRS in 1 of 2 studies. Seventy-nine percent of studies (n = 11 of 14) found increased frailty predicted prolonged hospital LOS, when employing the PSTFI, JHACG, mFI-11, HFRS, MSTFI (3 of 4 studies), and mFI-5 (2 of 4 studies). Eight of 9 studies found frailty predicts increased NRD rates, when employing the mFI-5, HFRS, JHACG, and MSTFI (1 of 2 studies). Only one of two studies employing the mFI-5, found increased frailty predicted an increased reoperation rate. Two of 3 studies found increased frailty predicted increased hospital readmission rates; increased MSTFI scores were associated with increased readmission in a single study, whereas mFI-5 predicted readmission in one of two studies. Only 27% (n = 6) reported the study cohort follow-up rate. No studies examined health-related quality of life, function, or cognition as clinical outcomes.

### Clinical Outcomes – Studies Employing Measures of Sarcopenia

Table 6 includes a summary of clinical outcomes stratified by sarcopenia measure. All studies employing sarcopenia measures examined mortality and/or overall survival. Of these studies, a single study examined the L4-PLVI in the context of predicting perioperative AEs. Eight studies examined sarcopenia measures in the context of postoperative AEs, 5 examined hospital LOS, 1 examined readmission rates, and 2 examined reoperation rates. The L4 CSMA/height^2^, L4-PLVI, L3-SMQ, L3-VAT, L4-VAT, and L4-SAT did not predict mortality (each examined in one study, respectively). The following measures of sarcopenia predicted mortality: L3-4-TPA (1 of 1 studies), L3-TPA/VBA (2 of 3 studies), L3 TPA/height^2^ (1 of 2 studies), BCA (1 of 1 studies), and L3-SAT (1 of 1 studies). Only the L3 TPA/VBA predicted postoperative AEs in a single study. The L3-4 TPA, L4 PLVI, BCA, L3-SMI, L3-SMQ, L3-VAT, and L3-SAT did not predict postoperative AEs (each in a single study). Of the studies examining hospital LOS, 4 did not find an association between increased sarcopenia and hospital LOS; a single study found low L3-SMQ was associated with increased LOS. Neither study examining reoperation and sarcopenia found a significant association with L3-4 TPA or L4-PLVI. Similarly, a single study examining readmission rates did not find an association with sarcopenia (L3-4 TPA). Only 27% (n = 6) examined the follow-up rate. No studies examined health-related quality of life, NRD, function, or cognition.

### Clinimetric Appraisal – Studies Examining Frailty Tools

The clinimetric properties of frailty tools are summarized in Table 7. The PSFTI, JHACG, mFI-11, MSTFI, mFI-5, and HFRS were found to be objective, but the JHACG was not. All tools were considered feasible, except the HFRS. PSTFI, JHACG, MSTFI, and mFI-5 tools lacked content validity, whereas the mFI-11 and HFRS had uncertain content validity. No tool was found to have construct validity. Only the HFRS had predicted validity in all studies employing it (n = 2; 100%). The mFI-5 had positive predictive validity in 2 of 4 studies and the MSTFI in only 2 of 7 studies. The mFI-11, PSTFI, and JHACG had either uncertain or negative predictive validity in all studies examining them, respectively. Neither reliability nor responsiveness were examined in any study. All frailty tools had either floor or ceiling effects. The most sensitive population for application of each respective frailty tool could not be determined given the clinimetric appraisal findings. All tools were studied in the context of risk stratification, and none were examined for their application toward assessing frailty trajectory.

### Clinimetric Appraisal – Studies Examining Sarcopenia Measures

Clinimetric properties of sarcopenia measures employed in spinal oncology populations are described in Table 8. No tools had construct validity, although the L3-TPA/VBA, L3-TPA/height^2^, L3-SMQ, L3-VAT, and L3-SAT had uncertain construct validity. Predictive validity was positive for the L3-TPA/VBA in only 1 of 3 studies. All other measures lacked predictive validity. The L3-TPA/VBA and L3-TPA/height^2^ were reliable. All other measures had uncertain reliability or were not assessed for this clinimetric property. Most studies did not assess responsiveness, although L3-TPA was found to have uncertain responsiveness. All sarcopenia measures were studied in the context of risk stratification. All measures were studied in the context of risk stratification, and none were examined for their application toward assessing sarcopenia trajectory.

### Study Bias Assessment

Study bias assignments are presented in Supplemental Content E. The most common sources of bias were related to inadequate follow-up duration for the outcome of interest to occur, no description of cohort follow-up duration (or rate), and analyses lacking adjustment for numerous pre-operative systemic variables known to influence post-operative clinical outcomes among patients undergoing surgery for spinal oncologic disease.

### Study Quality Assessment

All studies were graded either “Level 4” (n = 15; 68%) or “Level 2b” (n = 7; 32%) evidence quality according to the Oxford Levels of Evidence 2 grading scale for prognostic studies. Quality assignments are provided in Supplemental Content F.

## Discussion

### Summary of Findings

This systematic review summarizes measures of frailty and sarcopenia applied in surgical spine oncology and critically appraises their clinimetric properties using a validated set of qualitative criteria and definitions from the Consensus-based Standards for Health Measurement Instruments (COSMIN). No measure of frailty or sarcopenia had positive ratings for all properties, all lacked or had uncertain content and construct validity. Frailty tools had floor or ceiling effects. The MSTFI was studied most frequently but lacked predictive validity. The HFRS had positive predictive validity but lacked feasibility for use in the clinical setting. Most measures of sarcopenia lack predictive validity, and all required special tools or training for use. None of the frailty tools have been assessed for reliability, responsiveness, or used to track frailty trajectory. Here, we discuss the data and highlight clinimetric limitations of existing measures of frailty and sarcopenia. We also recommend a pragmatic approach to utilizing existing measures of frailty and sarcopenia in spinal oncology until more clinimetrically robust tools are developed.

### Towards the Development of a Clinimetrically Robust Measure of Frailty or Sarcopenia in Surgical Spine Oncology

We observed that all previously reported tools for assessing frailty were lacking or had uncertain content validity. This property may be optimized by including items that are comprehensible, relevant, and represent a comprehensive sample with respect to frailty in the setting of spine oncology.^28^ The AO Spine Knowledge Forum Tumor (AOSKFT) recently conducted a systematic review to identify which systemic variables influence post-operative outcomes in the context of spinal metastatic disease.^2^ Older age, low body mass index, comorbidity burden, non-ambulatory status, biochemical abnormalities, malnutrition, and systemic disease burden have been found to predict worse survival, increase adverse events, and worse health-related quality of life.^2^ None of the current frailty measurement include a comprehensive sample of these variables. Tools such as the mFI-5, mFI-11, MSTFI, and PSTFI predominantly assess comorbidity status but do not account for the physiological effects of systemic disease, tumor burden, or use of adjuvant therapies, which influence physiological reserve and the ability to tolerate surgery.^2,10,12,41^ The mFI-11 and mFI-5 include only a limited number of deficits, rendering them susceptible to instability and imprecise index estimates.^6^ The MSTFI includes case urgency and surgical approach, neither of which are factors related to patient frailty or would typically be included in a frailty index.^5,6^ The lack of predictive validity of the PSTFI was not surprising, given it was derived from the MSTFI, the values used to stratify frailty were arbitrarily chosen, and it predominantly assesses comorbidity status among patients that are typically younger, healthier, and have fewer comorbidities than those with spinal metastatic disease.

To better define the multidimensional nature of frailty in the surgical spine oncology context, the AOSKFT utilized a modified Delphi approach in combination with an international cross-sectional survey of the AO Spine community to assess perceptions of frailty in the context of spinal metastatic disease.^13^ Pre-operative systemic variables were ranked for their association with frailty using weighted analysis. Consensus (defined as >70% agreement among respondents) was attained among respondents regarding the association between 14 pre-operative clinical variables and frailty. Examples included high risk cardiopulmonary disease, renal failure, liver failure, malnutrition, systemic disease burden, poor performance status, recent unplanned hospitalization secondary to complications of the underlying cancer, and ongoing cytotoxic chemotherapy and/or targeted therapy. Most respondents indicated they evaluate frailty based on general clinical impression, highlighting the outstanding need for a practical and objective frailty assessment tool. Their findings could be used to improve content validity of a future tool for measuring frailty in this patient population.

We observed that most measures of frailty lacked predictive validity. Similar findings were reported by Moskven et al^12^ in their clinimetric appraisal of 14 frailty tools used in the setting of spinal surgery for degenerative or deformity-related conditions. Most tools had poor predictive validity, and no frailty tool received positive ratings for all clinimetric properties. The HFRS was found to be a validated risk stratification tool for predicting postoperative AEs following spine surgery for degenerative conditions. The HFRS has been used to predict AEs among patients with primary^47^ and metastatic spinal tumors.^45^ Although the HFRS has positive predictive validity, the 109-item tool lacks feasibility and could not be reasonably completed in clinical practice. Surgical decision-making particularly in the context of spinal metastatic disease often occurs after hours and on an urgent basis; a tool that can be quickly and easily applied is desirable. No study reviewed described the average time required to complete the frailty assessment. The MSTFI is the most frequently studied frailty tool in surgical spine oncology and is both feasible and objective; however, the predictive validity was positive in only 2 of 7 studies. Massaad et al^9^ found the MSTFI overestimated the risk of postoperative AEs among severely frail patients and underestimated risk among mildly frail patients. Bourassa-Moreau et al^10^ did not find the MSTFI predicts postoperative AEs. The construct validity, reliability, and responsiveness of both the HFRS and MSTFI remain unclear. Predictive validity was positive for the mFI-5 in 2 of 4 studies. All other frailty and sarcopenia tools had either uncertain or negative predictive validity.

Floor and ceiling effects were observed for all frailty tools. These effects limit the ability to differentiate patients with the lowest and highest scores and may partly explain poor predictive validity. Many existing frailty tools require only a single point to consider patients “pre-frail” or “frail”, resulting in a high cohort prevalence of frailty. Floor and ceiling effect can limit responsiveness (i.e. the ability of a tool to detect clinically important changes over time) and reliability (often measured with intraclass coefficient or weighted Cohen’s Kappa Coefficient analyses). Neither of these properties was directly examined for any frailty tool appraised. Moskven et al^12^ reported similarly that reliability and responsiveness were not examined for most frailty tools included in their review. Responsiveness may also be reduced by the modifiability of items included in each tool. Except for the HFRS, most frailty tools contained non-modifiable constructs. None of the frailty tools included in this review were used to track the trajectory of frailty or sarcopenia, rather they were retrospectively applied for the purpose of risk stratification and outcome prognostication.

Sarcopenia has been associated with unfavourable clinical outcomes in patients with cancer.^54,55^ For example, the L3-TPA/VBA and L3-TPA/Height^2^ have been utilized in oncologic research and their reliability has been demonstrated.^48,56,57^ However, the construct and predictive validity of these tools remains unclear in the context of surgical spine oncology. Bourassa-Moreau reported a positive association between L3-TPA/VBA and postoperative AEs among patients with spinal metastatic disease,^10^ however, predictive validity was unclear among all other studies reviewed. A potential explanation for these findings relates to traditional sarcopenia measurement techniques assessing psoas area, which may not represent total body sarcopenia.^19^ BCA techniques may offer better capture total body sarcopenia and elucidate the relationship between body habitus and clinical outcomes in the surgical spine oncology. First, they often include more robust measures of cross-sectional muscle, such as the skeletal muscle index (i.e. the collective psoas, paraspinal and abdominal muscle mass at a spinal level divided by height (cm^2^/m^2^)).^22^ Second, they may distinguish between distribution of muscle and adipose tissue, which may be differentially affected by adjuvant therapies.^20,21^ While muscle quantity has been associated with survival in cancer patients, muscle quality (e.g. radiodensity) has also been associated with survival, patients’ symptoms, and health care use.^58^ BCA has been applied in the surgical spine oncology setting. Massaad utilized an automated segmentation technique using validated machine-learning based pipelines based on convolutional neural networks, trained on abdominal CT data from large oncologic cohorts.^22^ In their retrospective analysis of institutional data, stratifying the cohort based on BCA phenotypes, the “high-risk” cluster had lower BMI, L3-SMI, SAT, VAT, and higher SMQ. In the analysis adjusted for age, sex, and frailty, there was an increased risk of death in the high-risk cluster (HR 1.45, 95% CI 1.05-2.01, *P* < .02). However, there was no difference in major AEs, LOS <7 days, or inpatient mortality between “low risk” and “high risk” clusters. Bongers et al^18^ separately reported a retrospective review of 196 patients undergoing surgery for spinal metastatic disease and found the L4 CSMA/Height^2^ was associated with 1 year, but not 90-day mortality. Abdominal adipose and muscle attenuation were not independently associated with mortality. Studies utilizing BCA remain sparse. Whether BCA offers an advantage for risk stratification and outcome prognostication compared to traditional psoas measurement techniques remains to be determined.

### Limitations

We recognize there are two study limitations that were introduced by our search strategy, including: (1) the use of an English language filter, which may have limited the inclusion of relevant evidence in other languages; and (2) our use of a date filter (2000-date of search), which was informed by our knowledge of frailty and sarcopenia tools in spinal oncology but may have omitted relevant evidence published prior to 2000.

### A Pragmatic Approach to Utilizing Existing Frailty and Sarcopenia Measures in Spinal Oncology: AO Spine Knowledge Forum Tumor Working Group Recommendations

1. Clinical decision making regarding the ability to tolerate palliative surgery should account for the following variables:
  (a) Systemic disease burden
  (b) Severe comorbidities (high risk cardiopulmonary, renal failure, liver failure, malnutrition)
  (c) Reduced performance status (KPS < 70)
2. Until a clinimetrically robust, disease-specific frailty assessment tool is developed, the mFI-5 may be considered as a decision-making adjunct, supplementing factors described in recommendation 1:

Rationale: The mFI-5 is objective, feasible, and predicts non routine discharge (4 of 4 studies), any AEs (3 of 3), increased hospital LOS (2 of 4), and mortality (1 of 1). However, this tool has questionable content validity (includes 4 comorbidities and dependent functional status), a floor effect, and responsiveness is unknown.

3. The L3-TPA/VBA has the most positive clinimetric properties among sarcopenia measures applied in spinal oncology to-date, but requires further study before it can be recommended for use toward risk stratification given the paucity of studies evaluating its contextual utility:

Rationale: The L3-TPA/VBA is objective, feasible, has established reliability in surgical oncology, and predicts mortality (2 of 3 studies) and AEs (1 of 1 studies) in surgical spine oncology. However, this measure has been examined in few studies and responsiveness has not been determined.

4. There is insufficient data to support a recommendation regarding:
  (a) use of frailty tools or sarcopenia measures for assessing HRQoL, cognition, or trajectory of frailty and sarcopenia following surgery
  (b) use of body-composition analysis techniques for risk stratification in spinal oncology

### Future Work

Future studies should conduct prospective external validation of frailty tools employed for the purpose of risk stratification and outcome prognostication in the surgical spine oncology setting. Several previously reported tools are composed of arbitrarily chosen items. While the MSTFI and PSTFI were developed as disease-specific frailty assessment tools, they lack key disease-specific variables. For example, the MSTFI does not capture systemic disease burden. Given the paucity of disease-specific frailty measures for use in spinal oncology, it is too early to determine whether disease-specific instruments may outperform general measures of frailty for risk stratification and outcome prognostication. Items included in future frailty tools may be drawn from those recently reviewed by the AOSKFT,^2^ and ranked via weighted analysis,^13^ to improve construct, content, and predictive validity.^6^ Clinically relevant outcomes for future study in the context of frailty and surgical spine oncology may include neurological recovery, change in performance status, time-to-event regarding mortality, health-related quality of life, function and cognition.^13^ Inclusion of modifiable items in frailty tools may allow testing of interventions to improve frailty in the pre-operative setting, or track frailty trajectory following intervention. In addition to the impact of surgery on frailty, the effect of spinal tumors and systemic disease on frailty remains unclear in this context. Standardizing cut-off thresholds for defining frailty in the context of future frailty indices may improve interpretability, consistency, and comparability across patient cohorts. All measures of sarcopenia identified required special measurement techniques or training for use. In most cases, this included volumetric image analysis capable software, PACS imaging systems. Future work should strive to improve the feasibility of sarcopenia measurement and BCA techniques, particularly through automation and use of machine learning or other artificial intelligence modalities. Collaboration with neuroradiologists may facilitate an improved understanding and appreciation for technical considerations pertaining to image acquisition, properties, and analysis.

## Conclusion

Existing tools for evaluating frailty and sarcopenia among patients undergoing surgery for spinal tumors have poor clinimetric properties. While acknowledging these limitations, we provide pragmatic recommendations for utilizing existing frailty and sarcopenia measures in spinal oncology populations, until more clinimetrically robust tools are developed.

## Supplemental Material

Supplemental Material - “A Critical Appraisal of the Application of Frailty and Sarcopenia in the Spinal Oncology Population” Supplemental Material for “A Critical Appraisal of the Application of Frailty and Sarcopenia in the Spinal Oncology Population” by Mark A. MacLean, Antoinette J. Charles, Miltiadis Georgiopoulos, Jackie Phinney, Raphaële Charest-Morin, Rory Goodwin, Ilya Laufer, Michael G. Fehlings, John Shin, Nicholas Dea, Laurence D. Rhines, Arjun Sahgal, Ziya Gokaslan, Byron Stephens, Alexander C. Disch, Naresh Kumar, John O’Toole, Daniel M. Sciubba, Cordula Netzer, Tony Goldschlager, Wende Gibbs and Michael H. Weber, on behalf of the AO Spine Knowledge Forum Tumor in Global Spine Journal.

## Appendix

### Abbreviations

ACS: American college of surgeons
AKI: Acute kidney injury
aOR: Adjusted odds ratio
ARDS: Acute respiratory distress syndrome
aRR: Adjusted relative risk
AUC: Area under the curve
AE: Adverse event
BMI: Body mass index
CI: Confidence interval
Clin Nutrition: Clinical nutrition
CSA: Cross sectional area
CSF: Cerebrospinal fluid
CSMA: Cross sectional muscle area
CT: Computed tomography
d: Day
DFS: Disease free survival
DVT: Deep vein thrombosis
ECOG: Eastern cooperative oncology group
ESJ: European spine journal
F/U: Follow-up
GCT: Giant cell tumor
GI: Gastrointestinal
GSJ: Global spine journal
HFRS: Hospital frailty risk score
HR: Hazard ratio
HU: Hounsfield units
ICU: Intensive care unit
J Clin Neurosci: Journal of clinical neurosciences
JHACG: Johns Hopkins adjusted clinical groups
JNS: Journal of neurosurgery
JSO: Journal of surgical oncology
LOS: Length of stay
M: Month
mFI: Modified frailty index
MPNST: Malignant peripheral nerve sheath tumor
MSTFI: Metastatic spine tumor frailty index
NE: Not examined
NIH: National institute of health
NIS: National inpatient sample
NR: Not reported
NSCLC: Non small cell lung
NSQIP: National surgical quality improvement program
OR: Odds ratio
OS: Overall survival
PACS: Picture archiving communication system
PE: Pulmonary embolism
PLVI: Psoas lumbar vertebral index
PSTFI: Primary spine tumor frailty index
ROC: Receiver operator characteristic
RR: Relative risk
SATA: Subcutaneous adipose tissue area
SD: Standard deviation
SMD: Spinal muscle density
SMI: Spinal muscle index
T: Tertile
TPA: Total psoas area
TSJ: The spine journal
UTI: Urinary tract infection
VATA: Visceral adipose tissue area
VBA: Vertebral body area
World Neurosurg: World neurosurgery.
