# Supplementary material for: A Critical Appraisal of the Application of Frailty and Sarcopenia in the Spinal Oncology Population
Source: Global Spine J. 2025 Jan 12;15(1 Suppl):47S–80S. doi: 10.1177/21925682231207325 (PMC11988247; doi:10.1177/21925682231207325)
Supplement: Supplemental Material - “A Critical Appraisal of the Application of Frailty and Sarcopenia in the Spinal Oncology Population” [file sj-pdf-1-gsj-10.1177_21925682231207325.pdf]

PRISMA 2020 Checklist

Section and Topic	Item #	Checklist item	Location where item is reported
**TITLE**			
Title	1	Identify the report as a systematic review.	N/A
**ABSTRACT**			
Abstract	2	See the PRISMA 2020 for Abstracts checklist.	Page 1
**INTRODUCTION**			
Rationale	3	Describe the rationale for the review in the context of existing knowledge.	Pages 2-3
Objectives	4	Provide an explicit statement of the objective(s) or question(s) the review addresses.	Page 3
**METHODS**			
Eligibility criteria	5	Specify the inclusion and exclusion criteria for the review and how studies were grouped for the syntheses.	Pages 3-4<br>Table 1
Information sources	6	Specify all databases, registers, websites, organisations, reference lists and other sources searched or consulted to identify studies. Specify the date when each source was last searched or consulted.	Pages 3-4<br>Supplemental Content A
Search strategy	7	Present the full search strategies for all databases, registers and websites, including any filters and limits used.	Pages 3-4<br>Supplemental Content B
Selection process	8	Specify the methods used to decide whether a study met the inclusion criteria of the review, including how many reviewers screened each record and each report retrieved, whether they worked independently, and if applicable, details of automation tools used in the process.	Page 4
Data collection process	9	Specify the methods used to collect data from reports, including how many reviewers collected data from each report, whether they worked independently, any processes for obtaining or confirming data from study investigators, and if applicable, details of automation tools used in the process.	Page 4
Data items	10a	List and define all outcomes for which data were sought. Specify whether all results that were compatible with each outcome domain in each study were sought (e.g. for all measures, time points, analyses), and if not, the methods used to decide which results to collect.	Page 4<br>Supplemental Content C
	10b	List and define all other variables for which data were sought (e.g. participant and intervention characteristics, funding sources). Describe any assumptions made about any missing or unclear information.	Page 4<br>Table 2
Study risk of bias assessment	11	Specify the methods used to assess risk of bias in the included studies, including details of the tool(s) used, how many reviewers assessed each study and whether they worked independently, and if applicable, details of automation tools used in the process.	Page 5<br>Supplemental Content E
Effect measures	12	Specify for each outcome the effect measure(s) (e.g. risk ratio, mean difference) used in the synthesis or presentation of results.	N/A
Synthesis methods	13a	Describe the processes used to decide which studies were eligible for each synthesis (e.g. tabulating the study intervention characteristics and comparing against the planned groups for each synthesis (item #5)).	Pages 3-5
	13b	Describe any methods required to prepare the data for presentation or synthesis, such as handling of missing summary statistics, or data conversions.	Pages 4-5
	13c	Describe any methods used to tabulate or visually display results of individual studies and syntheses.	Pages 4-5
	13d	Describe any methods used to synthesize results and provide a rationale for the choice(s). If meta-analysis was performed, describe the	Pages 4-5

For Peer Review

PRISMA 2020 Checklist

Section and Topic	Item #	Checklist item	Location where item is reported
		model(s), method(s) to identify the presence and extent of statistical heterogeneity, and software package(s) used.	
	13e	Describe any methods used to explore possible causes of heterogeneity among study results (e.g. subgroup analysis, meta-regression).	N/A
	13f	Describe any sensitivity analyses conducted to assess robustness of the synthesized results.	N/A
Reporting bias assessment	14	Describe any methods used to assess risk of bias due to missing results in a synthesis (arising from reporting biases).	Page 5
Certainty assessment	15	Describe any methods used to assess certainty (or confidence) in the body of evidence for an outcome.	N/A
**RESULTS**			
Study selection	16a	Describe the results of the search and selection process, from the number of records identified in the search to the number of studies included in the review, ideally using a flow diagram.	Pages 4-5<br>Figure 1
	16b	Cite studies that might appear to meet the inclusion criteria, but which were excluded, and explain why they were excluded.	Pages 4-5 and Figure 1
Study characteristics	17	Cite each included study and present its characteristics.	Page 5<br>Table 2
Risk of bias in studies	18	Present assessments of risk of bias for each included study.	Page 8<br>Supplemental Content E
Results of individual studies	19	For all outcomes, present, for each study: (a) summary statistics for each group (where appropriate) and (b) an effect estimate and its precision (e.g. confidence/credible interval), ideally using structured tables or plots.	N/A
Results of syntheses	20a	For each synthesis, briefly summarise the characteristics and risk of bias among contributing studies.	Pages 5-8<br>Tables 3-8<br>Supplemental Content D-E
	20b	Present results of all statistical syntheses conducted. If meta-analysis was done, present for each the summary estimate and its precision (e.g. confidence/credible interval) and measures of statistical heterogeneity. If comparing groups, describe the direction of the effect.	N/A
	20c	Present results of all investigations of possible causes of heterogeneity among study results.	N/A
	20d	Present results of all sensitivity analyses conducted to assess the robustness of the synthesized results.	N/A
Reporting biases	21	Present assessments of risk of bias due to missing results (arising from reporting biases) for each synthesis assessed.	N/A
Certainty of evidence	22	Present assessments of certainty (or confidence) in the body of evidence for each outcome assessed.	N/A
**DISCUSSION**			
Discussion	23a	Provide a general interpretation of the results in the context of other evidence.	Page 8
	23b	Discuss any limitations of the evidence included in the review.	Pages 9-12
	23c	Discuss any limitations of the review processes used.	Page 11-12
	23d	Discuss implications of the results for practice, policy, and future research.	Pages 11-12

For Peer Review

PRISMA 2020 Checklist

Section and Topic	Item #	Checklist item	Location where item is reported
**OTHER INFORMATION**			
Registration and protocol	24a	Provide registration information for the review, including register name and registration number, or state that the review was not registered.	Page 3
	24b	Indicate where the review protocol can be accessed, or state that a protocol was not prepared.	Page 3 and Reference Section
	24c	Describe and explain any amendments to information provided at registration or in the protocol.	N/A
Support	25	Describe sources of financial or non-financial support for the review, and the role of the funders or sponsors in the review.	Title Page
Competing interests	26	Declare any competing interests of review authors.	Page 13
Availability of data, code and other materials	27	Report which of the following are publicly available and where they can be found: template data collection forms; data extracted from included studies; data used for all analyses; analytic code; any other materials used in the review.	N/A

From: Page MJ, McKenzie JE, Bossuyt PM, Boutron I, Hoffmann TC, Mulrow CD, et al. The PRISMA 2020 statement: an updated guideline for reporting systematic reviews. BMJ 2021;372:n71. doi: 10.1136/bmj.n71

For more information, visit: http://www.prisma-statement.org/

For Peer Review

Search Strategies

Ovid MEDLINE (R) ALL <1946 to June 15, 2022>

1. Exp Spinal cord/
2. Exp Spine/
3. ((Primary OR Secondary OR Metasta* OR Tumour* OR Tumor* OR Oncolog* OR Cancer* OR Neoplasm*) AND (Spin*)).ti,ab,kf
4. Spinal Neoplasms/
5. Exp Spinal Cord Neoplasms/
6. 1-5/OR (362296)
7. Neurosurgery/
8. Neurosurgical procedures/
9. Surgical Oncology/
10. Surgical procedures, operative/
11. Exp Orthopedic procedures/
12. Neurosurg*.ti,ab,kf,in
13. Surg*.ti,ab,kf,in
14. Ortho*.ti,ab,kf
15. Exp Perioperative Period/
16. (Perioperative OR Intraoperative OR Postoperative).ti,ab,kf
17. 7-16/OR (4090594)
18. Frailty/
19. Sarcopenia/
20. Frail*.ti,ab,kf
21. Sarcopenia.ti,ab,kf
22. Geriatric Assessment/
23. (Geriatric* adj3 Assess*).ti,ab,kf
24. 18-23/OR (70524)
25. 6 AND 17 AND 24 (322)
26. Limit 25 to (English language and yr= “2000-Current”) (312)

Embase (Elsevier) (Date of last search: June 15, 2022)

1. 'spinal cord'/exp
2. 'spine'/exp
3. (primary:ti,ab,kw OR secondary:ti,ab,kw OR metasta*:ti,ab,kw OR tumour*:ti,ab,kw OR tumor*:ti,ab,kw OR oncolog*:ti,ab,kw OR cancer*:ti,ab,kw OR Neoplasm*:ti,ab,kw) AND spin*:ti,ab,kw
4. 'spine tumor'/exp
5. 'spinal cord tumor'/exp
6. #1 OR #2 OR #3 OR #4 OR #5 (455 347)
7. 'neurosurgery'/exp
8. 'surgical oncology'/exp

For Peer Review

9. 'surgery'/exp
10. 'orthopedic surgery'/exp
11. Neurosurg*:ti,ab,kw,ff
12. Surg*:ti,ab,kw,ff
13. Ortho*:ti,ab,kw
14. 'perioperative period'/exp
15. perioperative:ti,ab,kw OR intraoperative:ti,ab,kw OR postoperative:ti,ab,kw
16. #7 OR #8 OR #9 OR #10 OR #11 OR #12 OR #13 OR #14 or #15 (8 035 985)
17. 'frailty'/exp
18. 'sarcopenia'/exp
19. Frail*:ti,ab,kw
20. Sarcopenia:ti,ab,kw
21. 'geriatric assessment'/exp
22. (Geriatric* NEAR/3 Assess*):ti,ab,kw
23. #17 OR #18 OR #19 OR #20 OR #21 OR #22 (86 038)
24. #6 AND #16 AND #23 (1201)
25. #24 AND english:la AND [2000-2022]/py (1187)

Cochrane CENTRAL (Cochrane Library) (Date of last search: June 15, 2022)

1. [mh “Spinal Cord”]
2. [mh Spine]
3. ((Primary OR Secondary OR Metasta* OR Tumour* OR Tumor* OR Oncolog* OR Cancer* OR Neoplasm*) AND (Spin*)):ti,ab,kw
4. [mh ^“Spinal Neoplasms”]
5. [mh “Spinal Cord Neoplasms”]
6. #1 OR #2 OR #3 OR #4 OR #5 (21730)
7. [mh ^Neurosurgery]
8. [mh ^“Neurosurgical procedures”]
9. [mh ^“Surgical Oncology”]
10. [mh ^“Surgical procedures, operative”]
11. [mh “Orthopedic procedures”]
12. Neurosurg*:ti,ab,kw
13. Surg*:ti,ab,kw
14. Ortho*:ti,ab,kw
15. [mh “Perioperative Period”]
16. (Perioperative OR Intraoperative OR Postoperative):ti,ab,kw
17. #7 OR #8 OR #9 OR #10 OR #11 OR #12 OR #13 OR #14 OR #15 OR #16 (322297)
18. [mh ^Frailty]
19. [mh ^“Sarcopenia”]
20. Frail*:ti,ab,kw
21. Sarcopenia:ti,ab,kw
22. [mh “Geriatric Assessment”]
23. (Geriatric* NEAR/3 Assess*):ti,ab,kw

For Peer Review

24. #18 OR #19 OR #20 OR #21 OR #22 OR #23 (7894)
25. #6 AND #17 AND #24 (22 in CENTRAL)

Limits: 2000-Present

Note: Line result numbers are for all of the Cochrane Library, except line 25 which represents records pulled from CENTRAL only)

CINAHL with full text (EBSCO) (Date of last search: June 15, 2022)

1. (MH "Spinal Cord+")
2. (MH "Spine+")
3. ((TI (Primary OR Secondary OR Metasta* OR Tumour* OR Tumor* OR Oncolog* OR Cancer* OR Neoplasm*)) OR (AB (Primary OR Secondary OR Metasta* OR Tumour* OR Tumor* OR Oncolog* OR Cancer* OR Neoplasm*))) AND ((TI Spin*) OR (AB Spin*))
4. (MH "Spinal Cord Neoplasms")
5. S1 OR S2 OR S3 OR S4 (74,248)
6. (MH "Neurosurgery")
7. (MH "Oncology+")
8. (MH “Surgery, Operative”)
9. (MH "Orthopedic Surgery+")
10. (TI Neurosurg*) OR (AB Neurosurg*) OR (AF Neurosurg*)
11. ((TI Surg*) OR (AB Surg*) OR (AF Surg*))
12. ((TI Ortho*) OR (AB Ortho*)
13. (MH "Preoperative Period")
14. (MH "Intraoperative Period")
15. (MH "Postoperative Period")
16. ((TI (Perioperative OR Intraoperative OR Postoperative)) OR (AB (Perioperative OR Intraoperative OR Postoperative))
17. S6 OR S7 OR S8 OR S9 OR S10 OR S11 OR S12 OR S13 OR S14 OE S15 OR 16 (934 339)
18. (MH "Frailty Syndrome")
19. (MH "Sarcopenia")
20. ((TI Frail*) OR (AB Frail*)
21. (TI (Sarcopenia) OR (AB Sarcopenia)
22. (MH "Geriatric Assessment")
23. (TI (Geriatric* N3 Assess*)) OR (AB (Geriatric* N3 Assess*))
24. S18 OR S19 OR S20 OR S21 OR S22 OR S23 (33 209)
25. S5 AND S17 AND S24 (143)

Limits: English; 2000-Present (141 results)

For Peer Review

Data Extraction Elements

Demographic Data

1. Citation (including journal and year of publication)
2. Study Design
3. Database (if applicable)
4. Sample Size
5. Median or Mean Age (range, or SD if no range)
6. Age criteria (years)
7. % Male
8. Study population (primary, metastatic, both)
9. Primary Cancer Site (% by Type)
10. Frailty or sarcopenia tool examined

Frailty or Sarcopenia Tool Related

11. Component domains (e.g. comorbidities, laboratory, nutrition, radiographic)
12. Operational definition of frailty (e.g. cumulative deficit definition or weighted frailty models; whether scale is dichotomous or not; specify if has range of points)
13. Cutoff vaues (e.g. score, 1-2 pre frail, 3-5 frail, etc)
14. Number of items in tool
15. Setting of use and application
16. Special tools or training required for use
17. Prevalence of frailty and/or sarcopenia among cohort (overall, %)
18. Prevalence of frailty and/or sarcopenia among cohort stratified (by score, %)
19. Sarcopenia calculation

Clinical outcomes

20. Summary of primary study finding related to frailty or sarcopenia
21. Mortality (any time point; record time point)
22. Adverse events (major, minor, post-operative, peri-operative)
23. Length of stay
24. Hospital re-admission (rate)
25. Re-operation (rate)
26. Non routine discharge
27. Follow-up rate

Clinimetric (For definitions, please refer to Supplemental Content C)

28. Objectivity

For Peer Review

29. Feasibility
30. Content Validity
31. Construct Validity
32. Predictive Validity
33. Reliability
34. Responsiveness
35. Floor and/or ceiling effect
36. Current Clinical Application
37. Sensitive Population

For Peer Review

Frailty Tool Modifiability

Frailty Measure	Total Subscale Components	Domains	Items	Modifiable (M) vs Non Modifiable (NM)
PSTFI (Spinal Tumor Frailty Index)	9	Comorbidities	History of anemia	NM
			History of CHF	NM
			History of COPD	NM
			History of Pulmonary circulation disorders	NM
			Coagulopathy	M
			History of Renal failure	NM
		Radiographic Features	History of Pathological fractures	NM
		Nutrition	History of Malnutrition	NM
		Laboratory	Electrolyte abnormalities (any)	M
MSTFI (Metastatic Spinal Tumor Frailty Index)	9			
		Comorbidities	History of Chronic lung disease	NM
			History of pulmonary cirulation disorders	NM
			History of Renal failure	NM
		Nutrition	History of Malnutrition	NM
		Laboratory	History of anemia	NM
			History of Coagulapathy	NM
			Electrolyte abnormalities	M
		Surgical	Emergent/urgent case	NM
			Anterior or combined surgical approach	M
mFI (modified Frailty Index)	11	Comorbidies	History of diabetes mellitus	NM

For Peer Review

			History of COPD or pneumonia	NM
			History of congestive cardiac failure	NM
			History of an myocardial infarction	NM
			History of hypertension requiring medication	NM
			History of Peripheal vascular disease with revascularization or peripheral limb rest pain or gangrene	NM
			History of cerebrovascular accident or stroke with neurological deficit	NM
			History of percutaneous coronary intervention, cardiac surgery, stenting, or angina	NM
		Function	Partially dependent or totally dependent functional status	M
		Cognition	Impaired sensorium	NM
mFI-5 (modified Frailty Index)	5	Comorbidities	History of diabetes mellitus	NM
			History of hypertension requiring medication	NM
			History of CHF within 30d of surgery	NM
			History of COPD or pneumonia	NM
		Function	Dependent functional status	M
Hospital Frailty Risk Score	109	Comorbidities	Sequaele of cerebrovascular disease	NM
			Disorders of the urinary system (urinary tract infection and urinary incontinence)	M
			Haematuria	M
			Other bacterial agents as the cause of disease classified to other chapters	M
			Other cerebrovascular diseases	NM
			Convulsions, not elsewhere classified	NM

For Peer Review

			Somnolence, stupor, or coma	NM
			Complications of genitourinary prosthetic devices, implants and grafts	NM
			Other joint disorders, not elsewhere classified	NM
			Cellulitis	M
			Parkinson’s disease	NM
			Fractures of the ribs, sternum and thoracic spine	NM
			Other functional intestinal disorders	NM
			Acute renal failure	NM
			Decubitus ulcer	M
			Carrier of infectious disease	NM
			Streptococcus and staphylococcus as the cause of disease	NM
			Ulcer of lower limb, not elsewhere classified	NM
			Duodenal ulcer	NM
			Hypotension	M
			Unspecified renal failure	NM
			Septicaemia	M
			Personal history of other diseases and conditions	NM
			Respiratory failure, not elsewhere classified	NM
			Other arthrosis	NM
			Epilepsy	NM
			Osteoporosis without pathological fracture	M
			Fracture of femur	NM
			Fracture of lumbar spine and pelvis	NM

For Peer Review

			Other disorders of pancreatic internal secretion	NM
			Chronic renal failure	NM
			Other disorders of the kidney and ureter not elsewhere classified	NM
			Other degenerative disease of the nervous system, not elsewhere classified	NM
			Nosocomial condition	NM
			Other and unspecified injuries of head	NM
			Transient cerebral ischaemic attacks and related syndromes	NM
			Other soft tissue disorders, not elsewhere classified	NM
			Other bacterial intestinal infections	NM
			Diarrhea and gastroenteritis of presumed infectious origin	M
			Pneumonia, organism unspecified	M
			Aspiration pneumonitis	NM
			Gangrene, not elsewhere classified	NM
			Thyrotoxicosis (hyperthyroidism)	NM
			Scoliosis	NM
			Agent resistant to penicillin and related antibiotics	NM
			Osteoporosis with pathological fracture	NM
			Other disease of digestive system	NM
			Cerebral infarction	NM
			Calculus of kidney and ureter	NM
			Abnormalities of the heart beat	NM
			Unspecified lower respiratory tract infection	M
			Open wound forearm	M

For Peer Review

			Spinal stenosis	M
			Polyarthrosis	NM
			Other anemia's	M
			Other local infections of the skin and subcutaneous tissue	M
			Other non-infective gastroenteritis and colitis	NM
		Cognition	Dementia in Alzheimer's disease	NM
			Alzheimer's disease	NM
			Delirium, not induced by alcohol or other psychoactive substance	M
			Other signs/symptoms involving cognitive functions and awareness.	NM
			Senility	NM
			Unspecified dementia	NM
			Vascular dementia	NM
			Other signs/symptoms involving general sensations and perceptions	NM
		Falls	Hemiplegia	NM
			Other signs/symptoms involving the nervous system or musculoskeletal system which increase tendency to fall.	M
			Unspecified fall	NM
			Superficial injury to head	NM
			Abnormalities in gait and mobility	M
			Intracranial injury	NM
			Fracture of shoulder and upper arm	NM
			Other fall on same level	NM
			Superficial injury of lower leg	M
			Syncope and collapse	NM
			Falls involving bed	M

For Peer Review

			Open wound to head	NM
			Fall on and from stairs and steps	M
			Fall on same level from slipping, tripping and stumbling	M
		Function, Continency, and Visual-Audio Impairment	Care involving use of rehabilitation procedures	M
			Blindness and low vision	NM
			Abnormal results of function studies	NM
			Retention of urine	NM
			Unspecified urinary incontinence	NM
			Speech disturbances, not elsewhere classified	NM
			Other hearing loss	NM
			Dysphagia	NM
			Dependence on enabling machines and devices	M
		Laboratory	Disorders of fluids, electrolyte and acid-base balance	M
			Abnormal findings of blood chemistry	M
		Nutrition and Weight	Volume depletion	M
			Deficiency of other B group vitamins	M
			Vitamin D deficiency	M
			Disorders of mineral metabolism	NM
			Symptoms and signs concerning food and fluid intake	M
			Nausea and vomiting	M
		Mood	Symptoms and signs involving emotional state	M

For Peer Review

			Mental and behavioral disorders due to use of alcohol	NM
			Depressive episode	NM
		Social Support	Problems related to medical facilities and other healthcare	NM
			Problems related to social environment	M
			Problems related to care-provider dependency	M
			Problems related to life-management difficulty	M
		Other	Unknown and unspecified cause of morbidity	NM
			Artificial opening status	NM
			Other medical procedures as the cause of abnormal reaction of the patient	NM
			Personal history of risk-factors, not elsewhere classified	NM
			Fever of unknown origin	NM
Johns Hopkins Adjusted Clinical Groups (JHACG)	10			
		Comorbidities	History of decubitus ulcer	NM
		Cognition	History of dementia	NM
		Function	Urine incontinence	NM
			Vision impairment	NM
			Fecal incontinence	NM
		Falls	Difficulty walking	M
			History of fall	NM
		Socioeconomic	Poverty	M
			Barriers to care	M
			Lack of social support	M
		Nutrition	History of weight loss	NM

For Peer Review

			History of malnutrition	NM

For Peer Review

Newcastle Ottawa Risk of Bias Assessment

Study	Representativeness of exposed cohort	Selection of non exposed cohort or controls	Ascertainment of exposure	Demonstration that outcome of interest was not present at start of study	Comparibility of cohorts on the basis of the design or analysis	Assessment of outcome	Was followup long enough for outcomes to occur	Adequacy of followup of cohorts
Ahmed 2017	★	★	★	★	★	★	-	-
Bakhsheshian 2022	★	★	★	★	★★	★	-	-
Bongers 2022	★	★	★	★	★★	★	★	★
Bourassa Moreau 2020	★	★	★	★	★	★	-	-
Brinkmann 2021	-	★	★	★	★	★	★	★
Charest-Morin 2019	★	★	★	★	★★	★	-	-
De la Garza Ramos 2016	★	★	★	★	★	★	★	-
De la Garza Ramos 2021	★	★	★	★	-	★	★	-
Ehresman 2021	★	★	★	★	★★	★	★	★
Elsamadicy 2022 (JNS Spine)	★	★	★	★	★★	★	-	-
Elsamadicy 2022 (World NSx)	★	★	★	★	★★	★	-	-
Elsamadicy 2022 (Global Spine J)	★	★	★	★	★★	★	-	-
Gakhar 2015	★	★	★	★	-	★	★	★
Hersh 2022	★	★	★	★	★★	★	-	★
Hu 2022	★	★	★	★	★★	★	★	-
Kazim 2022	★	★	★	★	★	★	-	★
Lakomkin 2018	-	★	★	★	★	★	-	-
Massaad 2021 (Neurosurg Focus)	★	★	★	★	-	★	-	-
Massaad 2021 (J Neurosurg Spine)	-	-	★	★	★★	★	★	★

For Peer Review

Massaad 2022 (J Neurosurg Spine)	★	★	★	★	★★	★	★	-
Rothi 2019	★	★	★	★	-	★	★	-
Zakaria 2020	★	★	★	★	★★	★	-	-

For Peer Review

Oxford Center for Evidence Based Medicine Quality of Evidence Assessment

Ahmed 2017	4
Bakhsheshian 2022	4
Bongers 2022	4
Bourassa-Moreau 2020	4
Brinkmann 2021	4
Charest-Morin 2019	4
De la Garza Ramos 2016 (World NSx)	2b
De la Garza Ramos 2021 J Clin Neurosci	4
Ehresman 2021	2b
Elsamadicy 2022 J Neurosurg Spine	2b
Elsamadicy 2022 Global Spine J	2b
Elsamadicy 2022 World Neurosurg	2b
Gakhar 2015	4
Hersh 2022	2b
Hu 2022	4
Kazim 2022	2b
Lakomkin 2018	4
Massaad 2021 Neurosurg Focus	4
Massaad 2021 J Neurosurg Spine	4
Massaad 2022 J Neurosurg Spine	4
Rothi 2019 Journal of Clinical Neuroscience	4
Zakaria 2020 Neurosurgery	4

For Peer Review

Abbreviations

ACS – American College of Surgeons
AKI – Acute kidney Injury
aOR – adjusted odds ratio
ARDS – Acute Respiratory Distress Syndrome
aRR – adjusted relative risk
AUC – Area Under the Curve
AE – Adverse event
BMI – Body Mass Index
CI – Confidence Interval
Clin Nutrition – Clinical Nutrition
CSA – Cross Sectional Area
CSF – Cerebrospinal fluid
CSMA - Cross Sectional Muscle Area
CT – computed tomography
d – day
DFS – Disease Free Survival
DVT – Deep Vein Thrombosis
ECOG – Eastern Cooperative Oncology Group
ESJ – European Spine Journal
F/U – Follow-up
GCT – Giant Cell Tumor
GI - Gastrointestinal
GSJ – Global Spine Journal
HFRS – Hospital Frailty Risk Score
HR – Hazard Ratio
HU – Hounsfield Units
ICU – Intensive Care Unit
J Clin Neurosci – Journal of Clinical Neurosciences
JHACG – Johns Hopkins Adjusted Clinical Groups
JNS – Journal of Neurosurgery
JSO – Journal of Surgical Oncology
LOS – Length of Stay
M - month
mFI – modified Frailty Index
MPNST – Malignant Peripheral Nerve Sheath Tumor
MSTFI – Metastatic Spine Tumor Frailty Index
NE – Not examined
NIH – National Institute of Health
NIS – National Inpatient Sample
NR – Not reported
NSCLC – Non Small Cell Lung
NSQIP – National Surgical Quality Improvement Program

For Peer Review

OR – Odds Ratio
OS – Overall Survival
PACS – Picture Archiving Communication System
PE – Pulmonary Embolism
PLVI – Psoas Lumbar Vertebral Index
PSTFI – Primary Spine Tumor Frailty Index
ROC – Receiver Operator Characteristic
RR – Relative Risk
SATA – Subcutaneous Adipose Tissue Area
SD – Standard deviation
SMD – Spinal muscle density
SMI – Spinal muscle index
T - Tertile
TPA – Total Psoas Area
TSJ – The Spine Journal
UTI – Urinary tract infection
VATA – Visceral Adipose Tissue Area
VBA – Vertebral Body Area
World Neurosurg – World Neurosurgery

For Peer Review
